# Recent Findings on Thymoquinone and Its Applications as a Nanocarrier for the Treatment of Cancer and Rheumatoid Arthritis

**DOI:** 10.3390/pharmaceutics13060775

**Published:** 2021-05-22

**Authors:** Ravi Raj Pal, Vasundhara Rajpal, Priya Singh, Shubhini A. Saraf

**Affiliations:** 1 Department of Pharmaceutical Sciences, Babasaheb Bhimrao Ambedkar University (A Central University), VidyaVihar, Raebareli Road, Lucknow 226025, Uttar Pradesh, India; ravirajpal.rs@bbau.ac.in (R.R.P.); priyas.rs@bbau.ac.in (P.S.); 2 Department of Biotechology, Babasaheb Bhimrao Ambedkar University (A Central University), VidyaVihar, Raebareli Road, Lucknow 226025, Uttar Pradesh, India; vasundhararaj05@gmail.com

**Keywords:** thymoquinone, cancer, arthritis, nanotechnology, synovial delivery, toxicity reduction

## Abstract

Cancer causes a considerable amount of mortality in the world, while arthritis is an immunological dysregulation with multifactorial pathogenesis including genetic and environmental defects. Both conditions have inflammation as a part of their pathogenesis. Resistance to anticancer and disease-modifying antirheumatic drugs (DMARDs) happens frequently through the generation of energy-dependent transporters, which lead to the expulsion of cellular drug contents. Thymoquinone (TQ) is a bioactive molecule with anticancer as well as anti-inflammatory activities via the downregulation of several chemokines and cytokines. Nevertheless, the pharmacological importance and therapeutic feasibility of thymoquinone are underutilized due to intrinsic pharmacokinetics, including short half-life, inadequate biological stability, poor aqueous solubility, and low bioavailability. Owing to these pharmacokinetic limitations of TQ, nanoformulations have gained remarkable attention in recent years. Therefore, this compilation intends to critically analyze recent advancements in rheumatoid arthritis and cancer delivery of TQ. This literature search revealed that nanocarriers exhibit potential results in achieving targetability, maximizing drug internalization, as well as enhancing the anti-inflammatory and anticancer efficacy of TQ. Additionally, TQ-NPs (thymoquinone nanoparticles) as a therapeutic payload modulated autophagy as well as enhanced the potential of other drugs when given in combination. Moreover, nanoformulations improved pharmacokinetics, drug deposition, using EPR (enhanced permeability and retention) and receptor-mediated delivery, and enhanced anti-inflammatory and anticancer properties. TQ’s potential to reduce metal toxicity, its clinical trials and patents have also been discussed.

## 1. Introduction

As per the WHO, approximately 80% of the global population utilizes indigenous systems of medicine for their primary health care [1]. Recently, various potential phytocandidates such as β-elemene, brazilin, bufalin, cardamonin, cryptotanshinone, isogarcinol, curcumin, celastrol, lapachol, nobiletin, oroxylin A, thymoquinone, resveratrol, torilin, and swertiamarin have been identified to have pharmacological properties [2]. Thymoquinone (TQ) is a crucial active ingredient obtained from the black seed of the plant *Nigella sativa* (NS) and *Caramcarvil*, with potential antioxidant and anti-inflammatory activities [3]. It holds a wide range of other therapeutic properties, including hepatoprotective, cardioprotective, anticancer, antidiabetic, and antimicrobial properties [4]. Moreover, TQ also nullifies oxidative stress and prevents any damage to the tissue or cellular environment [5]. 

**Figure 1:**
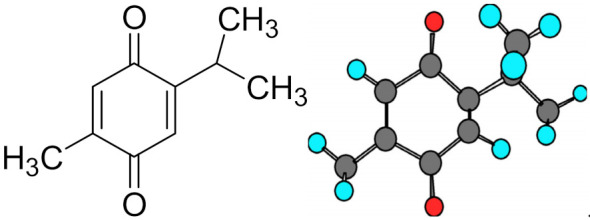
2D and 3D structure of thymoquinone, C_10_H_12_O_2._

The seeds of *N. sativa* contain a combination of volatile oils (0.40–0.45%), fixed oils (>30%, *wt/wt*) with two terpene alkaloids and eight fatty acids. Dithymoquinone, TQ, trans-anethol, (2-isopropyl-5-methylbenzo-1, 4-quinone), limonine, carvone, nigellidine, hedrin and p-cymene are some of the majorly identified terpenes. Moreover, the seeds also contain isoquinoline (nigellicimine-N-oxide and nigellicimine) and indazole alkaloids (nigellicimine and nigellidine) [6]. TQ exists in tautomeric forms in which the keto fraction (~90%) majorly exerts pharmacological actions [7]. The 2D and 3D structures of TQ are depicted in Figure 1.

TQ is a pharmacologically active agent used as a therapeutic agent as well as for preventive measures [8]. Oral dosing of *Nigella sativa* (NS) seeds at a quantity of 2 gm daily can effectively treat diabetes, as per reports [9]. However, it is associated with various pharmacokinetic issues that halt its pharmacodynamic activities. TQ is a hydrophobic molecule with low aqueous solubility and is associated with thermal instability and photosensitivity [10], which makes it systematically less bioavailable. Moreover, the bioavailability of TQ is mainly dependent upon its administration route. The absolute bioavailability (BA) of TQ in rabbits after oral (20 mg/kg PO) and IV (5 mg/kg) administration revealed a *58% lag time of 23 min with slower absorption and rapid elimination rates [11]. It is an acidic molecule with a pKa value of 5.1 [12] that is extensively degraded in the aqueous medium, especially at higher pH concentrations [1]. Low aqueous solubility, bioavailability, thermal, and photodegradability are some major drawbacks in utilizing its maximum potential as therapeutic. 

**Figure 2:**
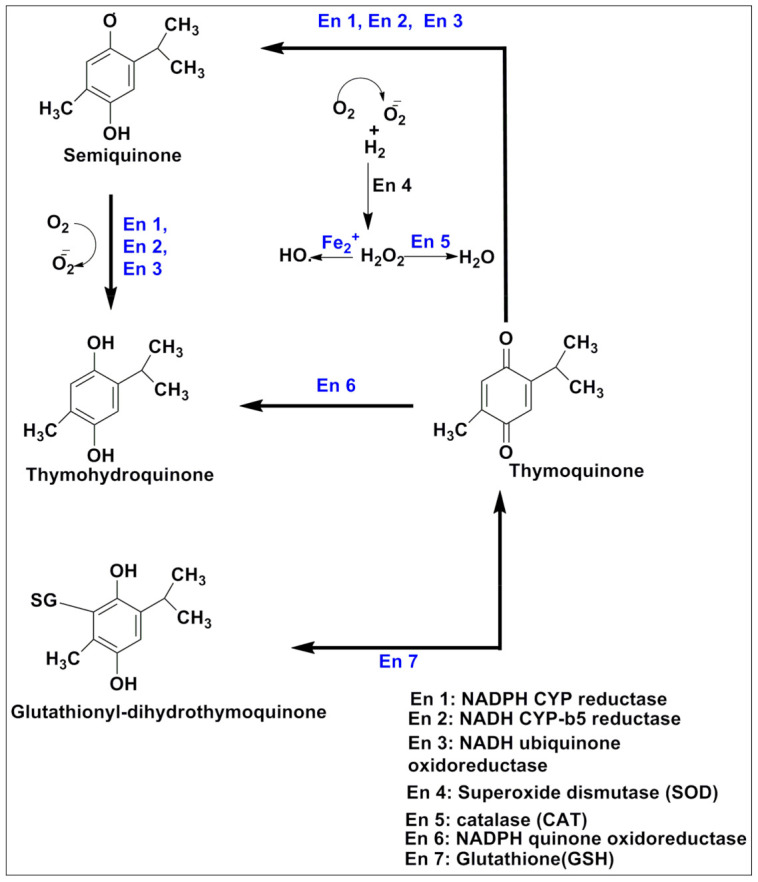
Enzymatic catalytic pathway of TQ under physiological conditions.

Orally administered TQ is biotransformed into hydroquinone by DT-diaphorase (a quinine reductase enzyme) [13]. Enzyme glutathione and NADPH (nicotinamide adenine dinucleotide phosphate oxidase) quinine oxidoreductase converted it into glutathionyl-dihydrothymoquinone and thymohydroquinone, respectively, via the redox mechanism [14]. TQ catalyzes in a two-step one-electron reduction or a two-electron one-step reduction. In one-electron two-step reduction of TQ, microsomal NADH cytochrome-b5 reductase, mitochondrial NADH ubiquinone oxidoreductase, and microsomal NADPH cytochrome P450 reductase convert TQ into semiquinone, which is further biotransformed into thymohydroquinone [15,16]. Conversely, a one-step two-electron reduction directs the conversion of TQ into thymohydroquinone [17]. Semiquinone of TQ is also known to possess oxidative stress-producing capabilities in cancerous tissues. Superoxide anion produced via oxidation can be nullified by TQ administration [18]. Due to the lack of detoxifying enzymes, which is quite common in cancer cells, the accumulated superoxide may exert the pro-oxidant effect of TQ [19]. The physiological catalysis of TQ is summarized in Figure 2. 

## 2. Method for Literature Search and Studies Selection 

The authors searched a number of electronic databases, namely Science Direct, Scopus, PubMed, US National Library of Medicine Clinical Trials (https://clinicaltrials.gov; accessed on 12 January 2021), and the Clinical Trial Registry of India (http://ctri.nic.in/; accessed on 12 January 2021). The following keywords were selected based on MeSH terms: thymoquinone, nanoparticle, nanocarrier, targeted nanoparticle, rheumatoid arthritis, nano, inflammation, cancer, neoplasm, toxicity, and antioxidant. These keywords were searched individually and in combination. At the first stage of screening, only English language articles were selected if the title, abstract, or full text contained the word “thymoquinone”. The initial database search found 5522 articles: 1389 from PubMed, 2191 from Scopus, 1933 from Science Direct, 7 from ClinicalTrials.gov, and 3 from the Clinical Trial Registry of India. In this process of analysis, 4240 articles were excluded due to them being indexed in two or more databases and were considered as duplicates. The remaining 1282 articles were screened out by analyzing the article’s title and abstract according to the inclusion criteria. After the second stage of screening, only 184 studies (158 experimental articles, 16 patents, 10 clinical txrials) were found to be appropriate according to the inclusion criteria. Studies of clinical trials in humans of any age, gender, or nationality, case–control studies, cohort studies, and randomized, double-blind, placebo-controlled, and parallel-group trials were considered for the review. Studies demonstrating the safety and efficacy of thymoquinone in in silico models were excluded for this review. Conference abstracts without full data or experimental information, letters to editors, and opinion papers, with potential influences of funding sources on the study results, were also excluded. The progression of thymoquinone articles from the year 2011 to 2021 is reported in Table 1. Furthermore, our review analysis indicated that TQ alone or in combination has beneficial roles in arthritic inflammation and various type of cancers. 

## 3. Rheumatoid Arthritis

Rheumatoid arthritis (RA) is recognized by the way the body’s immune system attacks the lining of the joints and results in significant mortality and morbidity rates. The lifetime risk of developing RA is increasing globally such as in the United States—1.7% (1 in 59) for men and 3.6% (1 in 28) for women; it varies within gender and individuals over time [20]. It is a long-lasting degenerative joint disease with an unknown etiology; however, it is thought to have multifactorial pathogenesis, including genetic and environmental defects as well as impaired immune regulation [21]. RA is characterized by joint inflammation, synovial membrane hyperplasia, excessive chemokines infiltration, leukocyte migration, and autoantibody production [22]. In the altered immune system, T cells fail to control inflammation and may initiate RA or other immune-related disease [23]. Additionally, the cell metabolism, whose primary work is to combat against the autoantigen attack, does not respond properly and effectively in RA conditions, which leads to chronic inflammation. Long-term RA inflammation alters cytokine release; overexpression of pro- and anti-inflammatory cytokines results in bone and cartilage damage. Patients with rheumatoid arthritis have unusual autoantibodies such as anticitrullinated antibodies and Rheumatoid Factor (RF), etc., that continue to circulate in the blood and thus, target their own body tissues, leading to polyarticular inflammation of the synovial membrane, wrists and feet along with nodule formation [24]. The synovium of RA is persistently upregulated by the induction of several chemokines and cytokines, including TNF-α, IL-1, IL-6, IL18, IL-15, and IL-12 [25]. Moreover, Toll-like receptors (TLR7, TLR4, TLR3, and TLR2) are also found to be upregulated in arthritic synovium [26] along with other inflammatory molecules, followed by the destruction of cartilage and bone [27]. Osteoclasts, the major bone resorbing cells, are mainly responsive to autoantibodies and inflammatory cytokines, in particular IL-1, IL-6 and TNF, which all induce osteoclast differentiation either directly or by inducing receptor activator of nuclear factor kappa B ligand (RANKL) activity [28]. Subsequently, stimulation of lymphocytes triggers cellular proliferation, differentiation, and also increased inflammatory cytokine synthesis (TNF-α, IL-7, and IL-1) [23,29]. In addition, RA patients also show pulmonary, cardiovascular, and other systemic complications [30]. 

The diversified pathogenesis of RA demands pharmacological and non-pharmacological approaches and sometimes, rotating interventions to achieve satisfactory therapeutic outcomes as well as patient compliance. Appropriate knowledge about the disease, preventive measures, optimized therapeutic regimens, and treatment goals for patients and health care providers might produce an appropriate impact upon RA amelioration.

Nevertheless, various non-pharmacological and pharmacological approaches, including recognition and avoidance of causative factors, non-steroidal anti-inflammatory drugs (NSAIDs), immunosuppressant therapies, herbal therapies (plant extract, oils), and physical measures (physiotherapy), have been used, either alone or in combination for the management of acute to chronic RA. The initial phase of RA can potentially be treated with NSAIDs; however, the chronic phase requires intensive therapies of disease modifying antirheumatic drugs (DMARDs), including modern biologics [31]. DMARDs such as methotrexate, leflunomide, sulfasalazine, and mycophenolate, etc., and modern biologics that specifically target cytokines and inflammation-inducing cells are used to ameliorate pain and prevent bone damage [31]. Besides their potential application in RA, DMARDs and biologics are also associated with numerous side effects such as TNF-α inhibition, which is associated with the risk of tuberculosis [32]; and tocilizumab, which is associated with a risk of lower intestinal perforation [33]. A large number of patients are resistant to current drugs with only 20–30% reaching low disease activity status and none of them can completely cure RA [31]. 

Currently, there is no absolute regimen for RA and cancer management owing to a multifaceted pathogenic interaction between a patient’s immunity, gene abnormality, and environmental susceptibility. Recently, bioactive compounds of natural origin such as thymoquinone and their nanoformulations were utilized for the treatment of cancer and rheumatoid arthritis. The potential of TQ and its nanoformulations-based targeted delivery for the management of cancer and arthritis is critically analyzed and reported in the following sections. 

### 3.1. Thymoquinone Works as an Anti-Arthritic 

**Figure 3:**
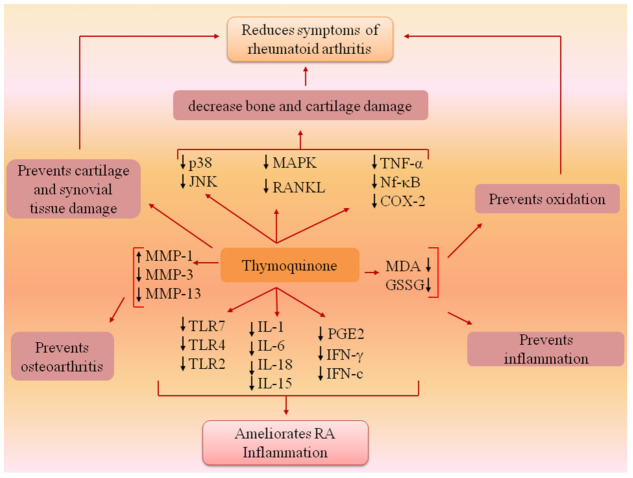
Anti-arthritic mechanism of TQ. TQ significantly downregulated the elevated levels of TLR-7, TLR-4, MMP-13, MMP-3, and other inflammatory cytokines, including TNF-α, IL-1β, PGE2, and IL-6 and upregulated the expression of MMP-1 to reduce arthritis scoring and bone leaching in arthritis. TLR—Toll-like receptor; IL—interleukin; PGE2—prostaglandin E2; MDA—malondialdehyde; GSSG—glutathione; MMP—matrix metalloproteinase; RANKL—receptor-activated nuclear factor kappa-B ligand; COX—cyclooxygenase-2; MAPK—mitogen-activated protein kinase.

Thymoquinone is a naturally occurring bioactive molecule reported to ameliorate rheumatic conditions in multiple pathways. TQ (10 mg/kg body weight) significantly downregulated the elevated level of Toll-like receptor (TLR) and other inflammatory cytokines (TNF-α, IL-1, and IL-6) in a Freund’s complete adjuvant (FCA)-induced arthritis rat in vivo model [34]. TQ (5 mg/kg body weight) significantly downregulated the level of pro-inflammatory mediators (IL-1b, IL-6, TNF-α, and prostaglandin E_2_) to reduce arthritis scoring and bone leaching in collagen-induced arthritis in a Wistar rats in vivo model [35]. In an in vivo study, Boudiaf et al. demonstrated that TQ (10–50 mg/kg, intraperitoneal) potentially inhibits N-formyl-methionyl-leucyl phenylalanine-induced neutrophil functions, and superoxide production [36]. In another in vitro study, the inhibition of phospho-p38 and phospho-JNK expression by TQ (0.1–5 μM) through apoptosis-regulated signaling kinase 1(ASK1) was reported to ameliorate rheumatic tissue damage [37]. Similarly, phosphorylation of p38 mitogen-activated protein kinase was blocked by TQ as investigated in both in vitro (isolated human RA fibroblast-like synoviocytes, dose 0–10 mM) and in vivo (rat adjuvant-induced arthritis, dose 5 mg/kg/day of TQ) studies; besides this, LPS-induced overexpression of inflammatory markers such as interleukin-1beta (IL-1b), TNF-α, cyclooxygenase-2, nuclear factor-kappa B-p65 metalloproteinase-13, and prostaglandin E_2_ (PGE2) were also regulated [8,38]. TQ decreases receptor-activated nuclear factor kappa-B ligand (RANKL)-induced osteoclastogenesis (in vitro in RAW 264.7 cells, TQ dose: 2.5, 5, and 7.5 µM) by inhibiting mitogen-activated protein kinase signaling and NF-κB (nuclear factor kappa light chain enhancer of activated B cells) as well as prevention of LPS (lipopolysaccharides)-induced bone erosion at a dose of 5 mg/kg as investigated in an in vivo C57/BL6 male mice model [39]. A similar potential of TQ (dose: 10 μM) in LPS-activated BV-2 murine microglial cells were also reported in an in vitro model [40]. TQ (intra-articularly injection of 0.3 mL; 10 mmol/L) also upregulated the expression of MMP-1 (matrix metalloproteinase-1) (tissue inhibitors) and downregulated MMP-13 in both rabbit chondrocytes and animal models of osteoarthritis induced by anterior cruciate ligament transaction [41]. The anti-inflammatory potential of TQ (dose of 2.5 mg/kg and 5 mg/kg) was found to be comparable with methotrexate (MTX) in an in vivo model of Freund’s incomplete adjuvant-induced arthritis [42]. Similar results were also observed to decrease carrageenan-induced inflammation in an in vivo rat model with an intraperitoneal dose of TQ (10 and 50 mg/kg) [36]. Moreover, the immunomodulatory effects of TQ (10 mg/kg of body weight, intraperitoneally) are almost similar to the therapeutic effects of MTX (0.5 mg/kg of body weight, intraperitoneally) as investigated in an FCA-induced arthritic in vivo model in rat [34]. An in vivo study by Pop et al. [43] has reported the anti-inflammatory and analgesic potential of NS oil in oral doses of 1, 2, and 4 mL/kg in comparison with diclofenac (5 mg/kg), as investigated in the carrageenan and Freund’s adjuvant-induced inflammatory in vivo model. In the same study, the antioxidant effects were studied and a decrease in malondialdehyde levels as well as oxidized glutathione was recorded. When caspase-1 cleaves, it leads to an increase in pro-inflammatory markers: for example, IL-1β, IL-18, post-NLRP3 (NOD-like receptor family pyrin domain containing 3) inflammasome activation [44]. TQ has been reported to block this cascade of events. [45]. The anti-arthritic mechanism of TQ is diagrammatically represented in Figure 3 and the applications of thymoquinone in the treatment of inflammation and arthritis are recorded in Table 2.

### 3.2. Encapsulated TQ Nanocarriers in the Treatment of Arthritic Inflammations 

The tumorigenic tissues and RA synovium exhibit likeness; for instance, EPR and hypoxia happen in both. The aim and strategies of nanoparticulate delivery for tumors can be similar to that of RA. The altered fenestrated synovial membrane in RA and tumor EPR could be a potential object for nanoparticulate-based drug delivery. The fenestrated and leaky vasculature of the synovial membrane favors penetration and retention of NP [47]. The nanocarriers have specific targeting ability to the inflammatory cells and thereafter, efficiently downregulate the pro-inflammatory sequence of events and can ameliorate RA indications and consequent bone damage; for example, macrophages increase at the arthritic inflammatory site and can engulf nanoparticles, resulting in passive targeting [48]. Moreover, NPs could also decrease the dose and off-target toxicities, thereby enhancing treatment potential for arthritic drug delivery [49,50]. To improve the stability and oral bioavailability of TQ, various nanoformulations, including an oral phospholipidic nanomatrix (particle size > 100 nm) [1], topical ethosomes (particle size 105.2 ± 8.0) [51], and liposomal chitosan gel [52], were developed which enhanced the therapeutic efficacy of TQ as investigated in a carrageenan-induced paw inflammation model. The phospholipidic nanomatrix made up of lipidic core and surfactant mixture enhances TQ aqueous solubility and intestinal absorption relative to TQ suspension [1]. Besides this, lipidic NPs are directly taken up by intestinal lymph and deliver the drugs directly into the bloodstream, which leads to avoidance of the first-pass metabolism process. As a result, lipidic NPs enhance the anti-inflammatory potential of TQ, vis TQ suspension as observed in the carrageenan-induced paw edema rat model. 

## 4. Neoplasm and Its Pathogenesis

A large group of individuals are diagnosed with cancer annually, being the second leading cause of mortality worldwide [53]. Its pathogenesis is very complex and is often difficult to identify, and most of the time, it is multifactorial. The tendency to multiply some groups of cells beyond their limit leads to abnormal development in a specific body part, which is called neoplasm or cancer [54]. Generally, metastasis-suppressor genes are involved in the inhibition of motility, invasiveness, colony formation, growth arrest, differentiation, proliferation, adhesion to extracellular matrix components, cell–cell adhesion and aggregation, and the immune sensitivity of cells [55,56]. All of these tasks require precise timing, which is controlled by a variety of cellular functions. Signaling, transcriptional activation, integrin expression and signaling, cell adhesion, and motility, cell communication, cytokine stress-induced signaling, serine protease expression, and nucleotide diphosphate kinase activity are among these functions [57]. Failing any of the above-said factor or group of factors may initiate cancer genesis [58]. Epigenetic changes also play a crucial role in disease initiation. Lower levels of H3K4me2, H3K18ac and H3K9me are linked to a poor prognosis in prostate, lung, and kidney cancers, respectively; similarly, higher levels of H3K9ac expression in lung cancer patients are linked to a shorter survival period [59,60]. Thymoquinone has recently been shown to modulate epigenetic machinery, such as histone acetylation and deacetylation, DNA methylation, and demethylation, all of which are significant epigenetic changes that may lead to carcinogenesis [61]. TQ has antineoplastic activity against human tumors, antioxidant effects and anti-inflammation in animal models and cell culture systems, chemopreventive effects, and most notably, anti-multidrug-resistant variants of human malignant cell [62]. 

### 4.1. The Mechanistic Approach to Treat Cancer Using TQ Drug Molecule 

The pharmacological effects of TQ on different cell lines and animal models demonstrated substantial antineoplastic activities in numerous cancers, including breast, prostate, brain, pancreas, gastric, colon, bladder, lungs, bone, cervical, and many more [63]. Mechanistically, it can suppress various properties, including multiplication in cancer cells, apoptosis, activation of detoxifying enzymes, metastasis, suppression of tumor-angiogenesis invasion, and cell cycle control [64,65,66,67,68,69,70,71,72]. 

Kinases are cellular enzyme stimuli, essential for cellular metabolic functions, and their overexpression is closely linked with cancer [73]. TQ effectively targets many phosphoinositides, including 3-kinase (PI3K) [74], mitogen-activated protein kinase (MAPK)/Janus kinase signal transducers and transcription (JAK/STAT) [75,76], polo-like kinase 1 (PLC1) [77] and tyrosine kinase [78]. 

**Figure 4:**
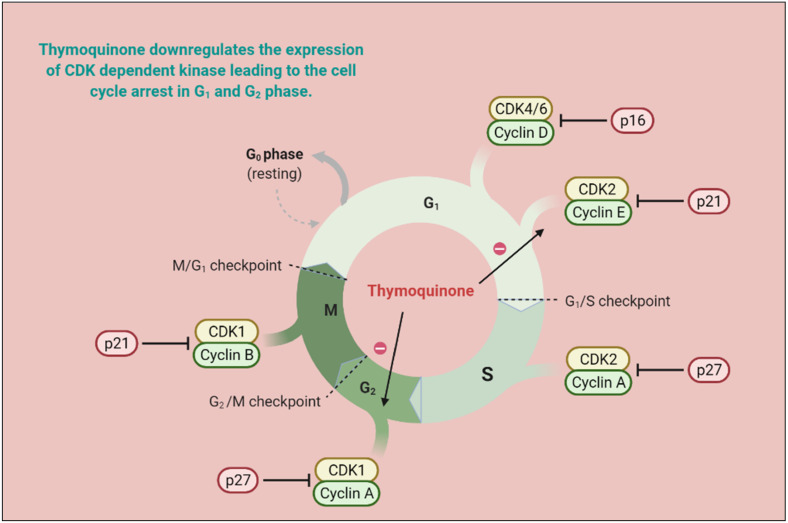
TQ prevents carcinogenic intermediate synthesis by inhibiting the G2/M phase of the cell cycle. It also inhibits ROS-mediated DNA damage to prevent tumorigenesis. TQ upregulates pro-apoptotic genes (p21 and p27) and downregulates the anti-apoptotic gene (Bcl-2), thereby arresting the G2/M phase of the cell cycle. (CDK—cyclin-dependent kinases; CYP—cytochrome P; TQ—Thymoquinone).

Responsive and resistive MCF-07 breast cancer cell lines displayed good anticarcinogenic activities with TQ analogs such as caryophyllyl and germacrylic conjugates as well as fatty acid conjugates [79]. The TQ neutralizes oxidative free radicals and ameliorates doxorubicin-induced nephrotoxicity [80]. The carcinogenesis produces eicosanoids, and peroxidizes membrane lipid suppressive activities [81]. Furthermore, TQ displayed a hyperproliferative effect in rats and also abrogated Fe (III) nitrilotriacetic acid (Fe-NTA) induced oxidative stress [63]. TQ reduced Cyclin A, Cyclin B1, Cyclin D1 and Cyclin E [82,83,84,85] expression and increased levels of p21 and p53 [86,87]. TQ is capable of decreasing Bcl-2 and increasing cleaved caspase-3, 9, and 7, and Bax proteins, as well as modulating the expression of microRNA (miRNA) and long non-coding RNAs (lncRNA), acetylation/deacetylation of histone along with methylation/demethylation of DNA, resulting in mitochondrial apoptosis induction [61,63,88,89]. TQ also halts the PI3K/AKT signaling pathway by upregulating PTEN, thus interfering with GSK-3β activity, enhancing β-catenin degradation, and decreasing MMP-9 and MMP-2 levels in esophageal cancer cells (Eca109 cells) [83]. MicroRNA-34a (miR-34a) expression is vital to cancer development and metastasis [90], and its expression is reduced by TQ in human metastatic breast cancers (MBC) compared to normal breast tissues [91]. Altogether, microRNA-34a can act as therapy either alone or in combination with TQ, and synergize therapeutic potential [92]. TQ exerts antiproliferative activities in cancer cells by modulating the structure of DNA [93,94]. TQ synergized pancreatic cancer cells (MIA Paca-2 cells) cytotoxicity along with juglone via ferroptosis, an iron-dependent mechanism [95]. The mechanistic approach of TQ for cancer treatment is depicted in Figure 4 and in vitro and in vivo applications of TQ are reported in Table 3 and Table 4, respectively.

### 4.2. TQ Nanocarrier for the Treatment of Cancer 

Many drugs do not reach the antineoplastic drug pipeline because of low aqueous solubility, high toxicity, large doses, and shorter half-life. Nanoformualtions provide opportunities to improve the pharmacokinetics of these drugs for precise treatment at the molecular level with reduced off-target effect [164,165]. The tumor tissues that exhibit enhanced permeability and retention (EPR) and hypoxia-like properties could be utilized for targeted drug delivery. The NPs take advantage of the EPR effect and accumulate in the cancer cells, providing maximum therapeutic efficacy with minimum off-target effect [166]. The nanoformulations, including polymeric (natural/synthetic), lipidic (liposomes, niosomes, ethosomes, cubosomes, solid lipid nanoparticles (SLN), nanoemulsion, and microemulsion), pretentious (bovine serum albumin, human serum albumin) and metallic (silver, gold, iron, etc.), in combination with surface modification, are utilized for targeted delivery of therapeutic drugs in tumor sites [167,168]. NPs deliver drugs at the selective tumor site utilizing multiple approaches, including passive targeting and active targeting. Some of them are explained in the following sections to deliver TQ at the target site. Applications of TQ nanocarriers and surface-modified TQ nanocarriers for the management of cancer and inflammation are reported in Table 5 and Table 6, respectively. Moreover, therapeutic importance of TQ-loaded nanoparticulate-based therapies for RA management is also reported in Table 5 with comparison to the conventional formulations and pure TQ.

#### 4.2.1. Passive Targeting Approach in Cancer Drug Delivery 

##### Passive Targeting Utilizes the Tumor Microenvironment for Drug Delivery

**Figure 5:**
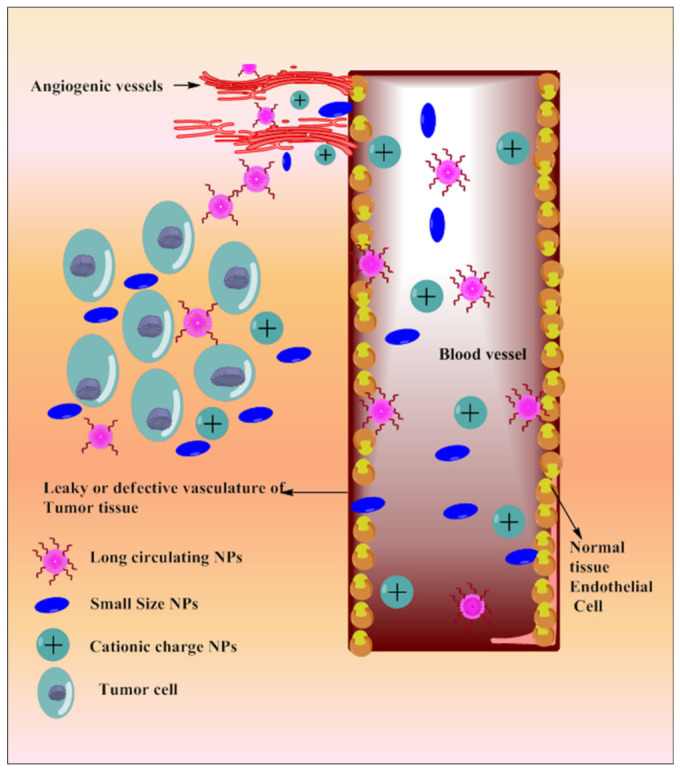
Systemic diagram depicting diverse approaches intended for passive targeting of TQ via nanoparticles.

Tumor vasculature is different from normal cell vasculature. Blood vessels of cancer tissue have comparatively larger fenestration with poor lymphatic drainage system, which results in enhanced retention and permeation of the nano-sized particulate matter [169]. Based on the delivery site, the size and surface of the NPs can be modulated. NPs’ size and surface architecture modulation also avoid reticuloendothelial system (RES) uptake and make it circulate for a long period of time. This could be explored in passive drug delivery. Various strategies depicting passive targeting of TQ via nanoparticles are reported in Figure 5. 

##### Passive Targeting through Long-Circulating Nanocarriers

Chitosan-grafted lipid nanocapsules [170] and PEGylated liposomes [171] were reported for the co-delivery of TQ and docetaxel (DTX) against drug-resistant breast cancer. Chitosan grafting improved cellular uptake and escaped endosomal effect; PEGylation increased circulation time of the dual payload [172], resulting in increased cytotoxicity against triple-negative breast cancer (TNBC) cells (MDA-MB-231 and MCF-7). A long-circulating PEGylated vitamin E lipidic nanocapsule loaded with TQ and DTX was also investigated against resistant breast cancer cells (MCF-7 and MDA-MB-231) [173]. PEGylation in vitamin E lipidic nanocapsules inhibits p-glycoprotein efflux, re-sensitizes the resistant TNBC cells and provides enhanced antimetastatic effects with reduced multiple side effects. Co-encapsulation of TQ with DTX improved loading efficiency into PEGylated liposomes and vitamin E lipidic nanocapsules as well as the chemosensitivity of DTX against breast cancer cells (MCF7 and MDA-MB-231).

PLGA-PEG-Pluronic TQ NPs were designed for sustained delivery of TQ into tamoxifen-resistant breast cancer cells (UACC 732, MCF-7) [174]. TQ-NPs reduce the dose and synergize tamoxifen chemoprevention potential with selective tumor cell toxicity. PEGylated LMW chitosan nanocapsules selectively deliver TQ into cancer cells (MCF 7 cells) [175] as chitosan (with pKa 6–6.5) solubilizes in the inter, as well as intracellular acidic microenvironment of cancer cells, thereby delivering TQ in a targeted manner.

##### Passive Targeting through Surface Charge and Size of NPs 

Nanocarriers overcome TQ pharmacokinetics issues and deliver it at the specific site with enhanced efficacy. A co-liposphere of Cabazitaxel (CBZ) and TQ was made of vitamin E-TPGS tricaprin, and egg phosphatidylcholine improved cellular internalization, which potentiates dose-dependent apoptosis as well as anticancer efficacy against MDA-MB-231 and MCF-7 cell lines [176]. The poly-L-lysine (PLL) and polyethylene glycol surface-decorated nanocontainers (NC-PLL) complex of diethylaminoethyl dextran/xanthan gum enhanced intracellular accumulation of TQ [177]. The positive surface charge of the NC-PLL significantly favored nanocontainer binding on the negatively charged cell membrane as compared to nonmodified nanocontainers, resulting in negatively charged NC-PEG. NC-PLL dominated in terms of cytotoxic efficacy, as investigated in MCF-7, likely due to enhanced accumulation in cancer cells.

Mesoporous silica NPs (TQ-MSNPs) improved TQ aqueous solubility and photostability as well as reduced the therapeutic dose (8-fold), which delayed cell migration and enhanced cytotoxic and apoptotic potential, as evaluated in the MCF-7 and HeLa cell lines [178]. The core-shell NPs of mesoporous silica delivered TQ to glioma cells selectively, which triggered cytochrome c, increased caspase-3 activation, and cell cycle arrest at the G2/M phase [179]. Chitosan-coated PLGA NPs containing TQ enhanced cytotoxic potential when compared with surface-decorated TQ-poly(lactic co-glycolic acid) NPs and TQ alone; this was investigated through the MDA-MB-231 and MCF-7 cell lines [180]. The antimetastatic potential of TQ was enhanced by chitosan nanoparticles against HepG2 cell lines through longer duration inhibitory actions when compared with free TQ [181]. TQ-NLC-NPs accumulated in cancer cells and inhibited their proliferation through time and dose-dependent modulation in the cellular morphology, as investigated in HepG2 cancer cells [182]. The polymeric NPs of methoxy poly(ethylene glycol)-b-poly(-caprolactone) improved the systemic bioavailability of TQ (1.3-fold) with slower elimination rates, which provides greater antiproliferative efficacy against varieties of pure cell cultures of human carcinoma (PANC-1, MCF-7, and Caco-2) [78,183]. The nanoarchitecture of polymeric shells increased TQ solubility, intestinal absorption, and bioavailability rates, resulting in higher cancer cell selectivity compared to free TQ. A soy phytosomal formulation of TQ with a dual release pattern (initial burst followed by prolonged release) revealed excellent anticancer activity against a lung cancer cell line (A539) [184]. The sustained release of TQ from phytosome accumulates TQ in the G2-M and pre-G1 phases of cancer cells, which initiate dose-dependent apoptosis and cell necrosis activities via caspase-3 activation. A Soluplus^®^-Solutol^®^ HS15 micelles formulation enhanced the antimigratory efficacy of TQ (1.5–10 µM) through improving aqueous solubility (10 times) and encapsulation efficacy, as investigated in SH-SY5Y human neuroblastoma cells [185]. The synergistic potential of TQ loaded in cockle-shell-derived aragonite CaCl_3_-NPs was reported with doxorubicin to reduce cellular migration in mammary gland carcinoma stem cells (MDA MB 231) [186]. A cubosomal formulation of TQ improved cellular accumulation, which leads to increased apoptotic activity migration in mammary gland carcinoma cell lines (MDA-MB-231 and MCF-7) [187]. Chitosan-coated TQ-PLGA-NPs accumulated in melanoma cancer cells (A375) by taking advantage of the EPR effect and positive surface charge of chitosan, which facilitate binding with the negatively charged cell membrane and induce cellular retention as well as time-dependent cytotoxicity [188]. TQ loading into niosomes improved cellular internalizations with controlled release of TQ, which markedly inhibits the migration of pro-inflammatory markers in mammary gland carcinoma with respect to pure TQ [10]. 

#### 4.2.2. Active Targeting 

##### Receptors Based Active Targeting 

**Figure 6:**
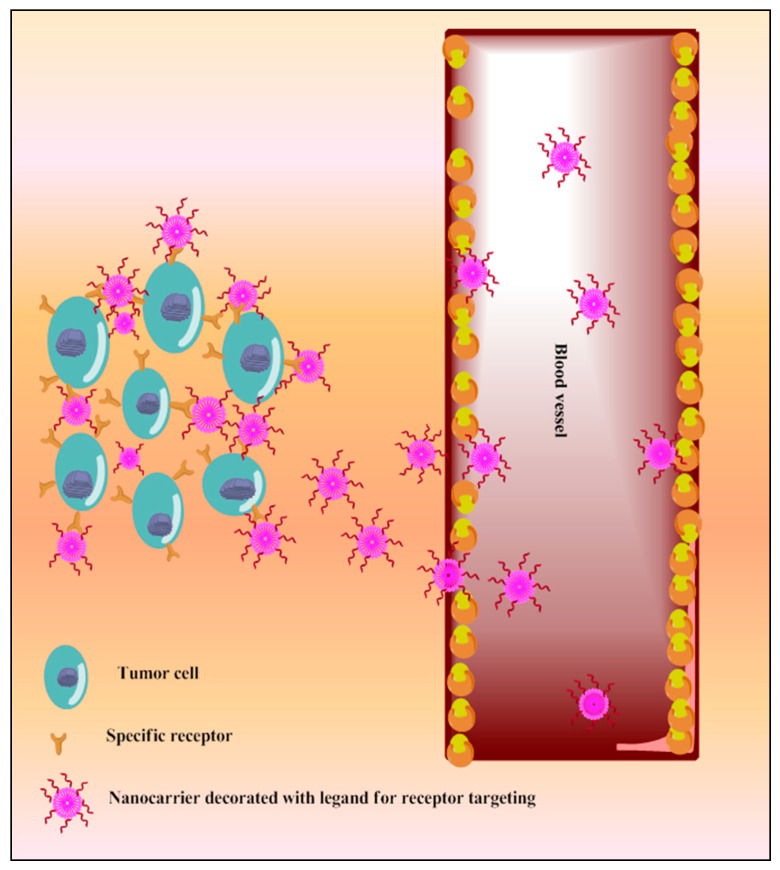
Schematic diagram of TQ nanocarriers for receptor-based active targeting.

A variety of surface receptors have been found to be upregulated in certain physiological conditions, including cancer, and are widely utilized for delivery via surface-decorated nanoparticles (NPs). The surface-coated NPs can target those cells which overexpress specific receptors on their surface and because of this, the nanoparticles attach to these [10]. The same is shown in Figure 6. The ligands which are used for surface modification include hyaluronic acid, anisamide, transferrin, folic acid, and many more utilized for active targeting of TQ into cancer. These have been reported in the following sections. This receptor is overexpressed in various types of cancers, including colon, brain, breast, lung, prostate, and kidney [189,190]. Anisamide is a benzamide analog, which exhibits a higher affinity towards sigma receptor-expressing cells [191] Anisamide-conjugated polymeric nanocapsules of eudragit-S100 delivered TQ into the colon-specific region through binding with overexpressed colonic sigma receptor [192]. The RNA aptamer, A10-coated planetary ball-milled starch NPs of TQ exclusively delivered drug into docetaxel-resistant prostate cancer cell lines (C4-2B-R and LNCaP-R) through overexpressed prostate-specific membrane antigen and inhibited drug efflux, which improves cancer potential [193]. The PEG and PCL, in the ball-milled NPs, decrease non-specific binding to the cell membrane and allow prolonged circulations. Hyaluronic acid (HA)-decorated Pluronic^®^ NPs of TQ accumulated in TNBC cells through selective binding with overexpressed CD44 receptor of cancer cells [194]. Pluronic-enhanced TQ encapsulation and HA facilitate CD44 targeting and make it have prolonged circulation, which reduced the dose for cell migration by modulating both miR-361/Rac1 and RhoA/actin stress fibers and the miR-361/VEGF-A mechanism that attenuate angiogenesis and metastasis of TNBC cells. Radio-iodinated NPs of folic acid-chitosan specifically bind to overexpressed folate receptors of human ovarian cancer cells (SKOV3) and improve anticancer efficacy through improved cellular internalization and retention [195]. A PEGylated-PLGA-TQ-NP surface decorated with transferrin potentiated anticancer efficacy of TQ through specific binding with the overexpressed transferring receptor on tumor cells, which decreases dose and improved cellular accumulations of NPs through EPR, as investigated in lung carcinoma A549 cells [196]. The as1411-conjugated nanodroplets delivered TQ into cancer cells through specific binding with overexpressed nucleolin on the cancer cells surface as investigated in MDA-MB-231 cells [197]. The as1411-conjugation facilitates rapid cellular uptake and dose-dependent cytotoxicity via nucleolin-stimulated Rac1 activation [198]. 

PI3K/Akt activation in cancer cells leads to resistance to traditional chemotherapeutics [199]. pH-sensitive gold niosomes of TQ along with Akt-siRNA were utilized to deliver TQ into tamoxifen-resistant breast cancer cells as well as knockdown of Akt-overexpression [96,200]. These niosomes resensitized cancer cells to TQ through Akt silencing and enhanced apoptosis by inhibiting MDM2 expression as well as inducing p53 [200].

##### Stimulus-Responsive NPs for Active Targeting 

Designing stimuli-responsive NPs for active targeted drug delivery is dependent upon tumor microenvironments such as pH, hyperthermia, catalytic enzymes, or external stimuli such as pressure, ultrasonication, or magnetic field. The stimuli-responsive NPs retain their physicochemical properties, including structure, during their circulation. They are stimulated upon exposure to small changes in the tumor microenvironment or external stimuli and undergo rapid changes (aggregation, permeability, and disruption) to release the encapsulated drug. Various TQ-loaded stimuli-responsive NPs with enhanced anticancer potentials have been discussed in the following sections. A TQ-loaded Fe_3_SO_4_ NPs surface decorated with ethylene glycol and polyvinylpyrrolidone (PVP) pH-dependently delivered TQ in TNBC cells (MDA-MB-231) [201]. PVP surface decoration improved water solubility and delivered drugs in the acidic environment, which maximized tumoricidal efficiency. 

Eudragit L-100-coated nanoconjugates of chitosan, HPMC, and PVA pH dependently delivered TQ into the colon for cancer management [202]. This study finds that at pH 7 concentration, eudragit L-100 dissolves and chitosan becomes degraded by anaerobic bacteria. The bacterial fermentation end-product butyrate forms polysaccharides with anticancer potential; TQ is released with butyrate and reaches into cancer cells, showing higher cytotoxicity. A technetium-99m (^99m^Tc)-labeled TQ formulation was designed for theranostic application against skeletal muscle malignancy (rhabdomyosarcoma) [203]. The ^99m^TC with TQ synergizes anticancer potential through rapid internalization and slower externalization, which enhanced theranostic applications. A fluorescent liposome co-delivered TQ and curcumin into lung cancer cells (A549) and potentially inhibited cellular proliferation compared with TQ or curcumin alone or the lipidic formulation of either of them, probably due to improved internalization [204]. A TQ-capped magnetic nanoparticle of iron oxide improved endocytotic internalization in breast cancer cells (MDA-MB-231 cells) and displayed a potent synergistic chemo-photothermal effect compared with free TQ [205]. Guar gum microvehicles rapidly release TQ in the intracellular acidic environment of cancer cells (pH~ 5.5) compared to physiological pH (~7.4), due to breakdown of the interlinking bonds in an acidic environment, leading to prolonged TQ release, with synergistic anticancer activity, as investigated in HepG2 cell line [206].

## 5. Role of TQ in Toxicity Reduction

TQ is systemically well-tolerated with a large safety profile dose (LD_50_, 2.5 g/kg) [3] and has the potential to reduce oxidative stress and systemic toxicity as the dose increases. The intravenous dose of 25 mg/kg thymoquinone nanostructured lipid carrier (TQ-NLC) was found safe in female Sprague Dawley rats [221]. It shows antiproliferative effect at 20 µM, genotoxicity at concentration ≥1.25 µM, and cellular narcosis at between 2.5 and 20 µM concentrations in the rat hepatocyte [222]. TQ (10 mg/kg) ameliorated sodium arsenate (20 mg/kg)-induced neurotoxicity by increasing the levels of norepinephrine, dopamine, superoxide dismutase, and catalase, and decreases serotonin, nitrate, and tumor necrosis factor alpha (TNF-α) levels in the cerebellum, cortex, and brain stem regions [223]. In another study, the neuroprotective effect of TQ (10 mg/kg/day) was observed on electromagnetic radiation-induced oxidative stress [224]. Similarly, glutamate and iron oxide nanoparticle-induced toxicity were also attenuated by TQ [5]. A combined formulation of *Costus speciosus*, *Fumaria indica, Cichorium intybus*, and TQ (CFCT) (25 mg/kg per oral) decreases cisplatin-induced hepatorenal toxicity in rats through membrane stabilization and decreasing aspartate aminotransferase, alanine aminotransferase, and alkaline phosphatase serum levels [225]. 

## 6. Recent Update on Patents of Thymoquinone

The latest patent literature search on thymoquinone and its loaded nanocarriers reported potential applications in the prevention, balancing, and treatment of multiple physiological conditions such as cancer, inflammations, dermal disorders, anxiety, and stress-related disorders; treatment of female urinary tract infections; and management of immunological diseases, etc. TQ was patented alone and in combinations for the treatment of inflammatory symptoms, including the eicosapentaenoic acid pathway [226]. Additionally, TQ and H5WYG peptide-loaded nanomicelles were also patented for targeted cancer drug delivery [227] and TQ-loaded nanodroplet emulsions for cancer targeting [228]. TQ-loaded nanocarriers are not limited to cancer targeting. Aminoglycoside-thymoquinone-loaded nano-liposomal formulations have been patented for aminoglycoside antibiotic delivery [229]. Authors rightfully assume an increase in patent outcomes when pure thymoquinone is converted to nanocarrier-loaded thymoquinone for various pharmacological applications. The patents illustrating the pharmacological significance of thymoquinone and related nanocarriers are recorded in Table 7.

## 7. Clinical Trials OF Thymoquinone

TQ has the potential to correct various physiological conditions of the body. It is widely investigated from dietary supplementation to chemoprevention. To date, a total of 10 clinical trials (Table 8) of thymoquinone claiming its effect on malignant lesions, aphtha, chronic periodontitis, type 2 diabetes mellitus, oral submucous fibrosis, pediatric major thalassemia, and supportive care in patients with COVID-19 are ongoing worldwide, the details of which are mentioned in Table 8. Moreover, recently, a clinical trial of TQ was registered to analyze efficacy and safety for best supportive measures (Guidelines on Clinical Management of COVID-19 issued by MOHFW, India) against COVID-19 patients. The confirmed COVID-19 patients were assigned as Cohort A and Cohort B. Cohort A patients received 50 mg TQ once a day for 14 days along with the best supportive measure, while Cohort B patients received the best supportive measure only. The trial was primarily evaluated for virologic (change in positive COVID-19 status on days 8 and 15) and clinical outcomes (proportion of patients on WHO progression scale 0 to 10 on days 8 and 15). A human trial (CTRI/2020/12/029514) of TQ tablets (dose of 50 mg; 25 mg; 12.5 mg) was registered to measure safety and tolerability and to analyze pharmacokinetic behavior in normal healthy adults under fasting conditions. A trial (NCT04686461) of thymoquinone extract is underway to investigate the effects against arsenical keratosis. In this trial, TQ-loaded topical ointment was used to treat 34 patients with arsenical keratosis at two-week intervals. The TQ ointment formulation was found to reduce the keratotic nodular size as well as improvement of the lesion calculated using the Likert Scale.

## 8. Conclusions and Prospects

TQ is a molecule that has multifaceted modes of action, including anti-arthritic and antineoplastic activities through modulating inflammatory and apoptotic pathways. However, its biological instability, rapid metabolism, poor water solubility, narrow bioavailability, inadequate cellular availability, and lack of targeting halt its transition from research to clinical application. Extensive literature analysis revealed that nanotechnology upgraded drug delivery patterns in cancer and arthritic disease through significant improvement in pharmacokinetics and target-oriented active molecules delivery, while decreasing their off-target side effects. To maintain the biological stability of TQ during formulation design or delivering alone, site-specific availability is among the major challenges to utilizing its maximum therapeutic potential in arthritis and cancer management. 

The role of TQ individually and its diverse types of nanoformulations for targeted delivery to tumorigenic cells and synovial tissues, with longer circulating time and higher synovial accumulation, improved anti-inflammatory and anticancer potential. The nanoformulation delivery of TQ results in significantly enhanced targeting payload and promising upgrades to its anti-inflammatory and anticancer efficacy. 

Nanoparticles are emerging carrier systems for the delivery of a wide range of therapeutic molecules. NPs are extremely attractive due to their important properties (size surface area and charge). Their use, as a drug carrier system or in theranostic applications including personalized medicine, might pave the way for a future strategy of prevention and counteraction of multiple diseases. 

In this review, we vitally analyzed and reported the possible mechanistic approach of thymoquinone, such as the downregulation of various cytokines, inflammatory factors, and apoptotic pathways for the management of rheumatoid arthritis and cancer. Moreover, their toxicity reduction potential was also reported. An extensive review of their patent and clinical trials worldwide was also reported. 

With the deep dive that we undertook in this review, it was revealed that formulations can transform the applicability of the nanocarrier-based formulation of thymoquinone; however, these studies can be dynamic. Significant dots in research have been recognized that need to be connected: various pre-clinical and human trials are taking place worldwide to ascertain the applicability of thymoquinone in humans; there are a lack of comparative findings on various nanoformulations to optimize the best regimen for TQ delivery against rheumatoid arthritis and cancer; the nonavailability of toxicity/safety data for thymoquinone-loaded NPs and human studies specifically exploring the pharmaceutical importance of nanoparticulate systems on arthritic and cancer milieu.

## Figures and Tables

**Table 1:** Progression of thymoquinone article from the year 2011 to 2021.

Database	Year Wise Progression of Articles										
	2021	2020	2019	2018	2017	2016	2015	2014	2013	2012	2011
PubMed	87	172	140	157	118	130	111	98	78	65	64
Scopus	121	285	201	212	203	177	155	127	78	96	89
Science Direct	177	277	188	202	158	128	106	112	78	88	54

**Table 2:** Applications of thymoquinone in the treatment of inflammation and arthritis (↓: decrease, ↑: increase).

S.N	Dose and Route	Animal Model/Cell Line	Molecular Target	Outcome	Reference
1	TQ (2–5 mg/mL/kg)	Pristane induced RA in female SD rats	↓IL-1β, ↓TNF-α, ↓IL-6, ↓IL-10, ↓IFN-γ, and ↓PGE2	RA amelioration	[7]
2	TQ (10 mg/kg BW)	Freund’s complete adjuvant (FCA) induced RA in rat	↓TLR2, ↓TLR4, ↓TNF-α, ↓IL-1, and ↓NF-κB	RA amelioration	[34]
3	TQ (1–5 μM)	TNF-α-induced synovial fibroblast activated RA	↓phospho-p38 and phospho-JNK expression through apoptosis regulated signaling kinase 1	↓tissue damage	[37]
4	TQ (10 and 50 mg/kg, Intrapleural)	pleurisy induced by λ-carrageenan in rats	impaired the phosphorylation on Ser-304 and Ser-328 of p47*PHOX*, a cytosolic subunit of the NADPH oxidase; ↓fMLF-induced neutrophil functions	↓of neutrophil accumulation in the pleural space, produce an anti-inflammatory response	[36]
5	5 mg/kg, oral	Nf-κB induced osteoclastogenesis (in vitro) and LPS induced bone loss (in vivo)	↓Nf-κB and MAPK, ROS, ↓c-Fos, and NFATc1	↓osteoclastogenes ↓bone loss	[39]
6	5 mg/kg, oral	Collagen-induced RA in rat	↓IL-1b, IL-6, TNFα, IFN-c, and PGE2	↓arthritis scoring and bone restoration	[35]
7	TQ 5 mg/kg oral	Isolated human fibroblast; adjuvant-induced RA	↓IL-1β, TNFα, MMP-13, cyclooxygenase-2, and PGE_2_, ↓phosphorylation of p38, MAPK, and NF-κB -p65	↓RA pathogenesis	[38]
8	Intra-articularly of 0.3 mL of TQ (10 µmol/L)	Anterior cruciate ligament transaction induced OA	↓MMP-3, MMP-13 ↑MMP-1 expression	Attenuated osteoarthritis (OA)	[41]
9	TQ (2.5–5 mg/kg)	FCA induced RA	↓IL-1β, ↓TNF-α	↓RA symptoms	[42]
10	TQ (0.1–100 µM)	Simpson–Golabi–Behmel syndromehuman pre-adipocytes.	↓IL-6, ↓IL-1β	↑antioxidant and ↑anti-inflammatory potential	[46]

Abbreviations: TQ—thymoquinone; SD—Sprague Dawley; OA—osteoarthritis; MMP—matrix metalloproteinase; OA—osteoarthritis; TLR—Toll-like receptors; PGE2—Prostaglandin E2; FCA—Freund’s complete adjuvant; IL—Interleukin; TNF-α—Tumor necrosis factor alpha; NF-κB—Nuclear factor kappa light chain enhancer of activated B cells; IFN-γ—Interferon gamma; MAPK—Mitogen-activated protein kinase; NFATc1—Nuclear factor of activated T-cells cytoplasmic 1; ROS—reactive oxygen species; JNK—c-Jun N-terminal kinase; NADPH—Nicotinamide adenine dinucleotide phosphate oxidase; fMLF—N-Formylmethionine-leucyl-phenylalanine].

**Table 3:** In vitro applications of thymoquinone in the treatment of cancer (↓: decreases, ↑: increase).

S.N	Drug and Dose	Cell Line	Molecular Target	Outcome	Ref.
1	TQ (25–75µM)	Eca109 cells	↑p21, and p53 levels; ↓Cyclin A, Cyclin B1, and Cyclin E expression; ↑β-catenin degradation, and ↓MMP-2, 9 levels; ↓in Bcl-2 and ↑caspase-3,7 and 9 cleavages, ↑Bax, ↑PTEN	Induced cell cycle arrest in the G2/M phase; ↓cell proliferation and invasion	[83,96]
2	TQ (511.19 µM) and juglone (40.90 µM)	MIA PaCa-2, BXPC-3, and Panc-1 pancreatic cancer cells	Ferroptosis	Synergism in anticancer potential	[95]
3	TQ (2.5–200 μM)	C6 rat glioma cells	Induced DNA damage, apoptosis, and ↑iROS. ↓GSH; ↑intracellular calcium level which initiates apoptosis ↓Bcl-2 and pSTAT3; ↑Bax, ↑Caspase-3,9; ↓MMP and GSH levels	Dose-dependent apoptosis induction	[97]
4	TQ (1–50 µM)	MDA-MB-231, MDA-MB-436, and BT-20	↓expression of eEF-2K, Src/FAK, and Akt; ↓NF-κB/miR-603 signaling axis	Dose-dependent ↓cell proliferation and migration	[98]
5	TQ, artemisinin hybrids	CCRF-CEM and Multidrug-Resistant CEM/ADR500 Leukemia Cells	Specifically inhibit cancer cells	Low toxicity/high selectivity profile	[99]
6	TQ(5µg/mL) and Emodin (25µg/mL)	MCF-7, MDA-MB 231, MDA-MB 468 and T47D	↑ROS generation; ↓FAK and Integrins, ↑p53, ↑Bax, and ↑cleaved caspase 3 expressions; ↓Bcl-2	↑apoptosis, ↓cell migration, and ↓stemness efficiently in breast cancer	[100]
7	TQ, TQ+cisplatin TQ+DOX	HCC HepG2 and SMMC-7721 HL-7702 cells	↑ROS, ↑caspase 3	↑apoptosis and selectively ↓cell viability	[101]
8	TQ (2–150 μM)	A375, B16F10	↓NLRP3 (NACHT, LRR, and pyrin domain-containing protein 3); ↓proteolytic cleavage of caspase-1; ↓IL-1β and ↓IL-18, ↓NF-κB, ↓ROS	Inactivation of caspase-1, ↓melanoma cells migration	[102]
9	TQ 20 gm/kg	HCT116	↓CD44, ↓EpCAM, ↓Ki67, ↑p53, ↑p21, ↓PCNA, ↑TUNEL positivity, ↓γ-H2AX	↓viability of 5FU-sensitive and resistant HCT116	[103]
10	DOX, TQ, TQ/DOX	HepG2, Huh7	↑miR-16 and miR-375, ↑caspase 3; ↓Bcl-2	↓apoptosis; ↓cell viability	[104]
11	TQ, cisplatin, geraniol	MCF-7	↑SOD, ↓myeloperoxidase, ↓lipid peroxidation; ↓8-isoprostane levels	↓cisplatin neurotoxicity	[105]
12	TQ (8 μM)	HEp-2	↓MMP; ↓mitochondrial cytochrome c release	↑apoptosis of tumor cells	[106]
13	TQ (20 mM or 40 mM)	Human glioblastoma cells T98G and U87MG, Gli36DEGFR	↑recruitment and accumulation of the microtubule-associated protein light chain 3-II (LC3-II); accumulation of the LC3-associated protein p62	↑autophagy and induces cathepsin-mediated, caspase-independent cell death	[107]
14	TQ (10–40 mM))	HaCaT, HEK001 HeLa	↑GSN levels, ↑p27, ↑cleaved PARP; ↑UHRF1 by HPV E6/E7 causes GSN silencing	↑apoptosis and cell cycle arrest in early stage	[108]
15	Indirubin-3-monoxime and TQ	A549	↓Bcl-2/Bax ratio, ↓p-AKT, ↓p-mTOR, ↓Caspase-3, ↓p-53, ↓NFκB, ↓Akt/mTOR/NFκB, ↑p38, ↑ROS; ↓tumor growth by targeting NF-κB; ↑PPAR-γ activation; ↓Akt, 4E-BP1, ↓eIF4E, S6R and ↓p70S6K phosphorylation	↓metastasis, ↑cell cycle arrest; ↓tumor growth	[85,109]
16	TQ (5 μM-10 μM)	clone E6-1, HL-60, K-562	↑thymine glycol metabolite; induce DNA damage; ↓guanine levels	↑antiproliferation, ↑apoptosis	[110]
17	TQ (10 μM)	OVCA429, SKOV3, HeyA8, OVCAR3, OVCAR8	↓JNK, ↓Src, ↓FAK are involved in LPA-induced invasive cell migration	↓migration of cancer cells in a dose-dependent manner	[111]
18	TQ (20- 40 μmol/L)	T24, 253J SV-HUC-1	↓activation of Wnt/β-catenin signaling pathway, ↑E-cadherin, and ↓N-cadherin, ↓vimentin, ↓MYC, ↓Axin-2, ↓MMP7, ↓CyclinD1, ↓β-catenin	↓epithelial–mesenchymal transition in bladder cancer cells	[112]
19	TQ (5 μM) and alpha-hederin (50 μM)	PC3, HT-29, HCT116	Zinc level modulations	Dose-dependent cytotoxicity	[113]
20	TQ (1–100 μM)	786-O cells	↑sub-G1 population and % of apoptotic cells. ↓collective migration	Induces dose and time-dependent cytotoxicity, ↓invasive potential	[114]
21	TQ and paclitaxel	MCF-7, T47D	↑Pre-G phase cells, ↓TWIST-1 gene, and ↑SNAIL-1, ↑SNAIL-2 genes.	↓paclitaxel resistance, ↑apoptosis, ↑necrosis,	[115]
22	TQ (50 µM), Cur (15 µM), Caff (10 mM), DOX	HCT116, MCF7	↓bromodeoxyuridine incorporation, ↑accumulation of senescence-associated β-galactosidase (SA-β-gal), ↑cell cycle arrest, and ↑p53, ↑P-p53, and ↑p21 proteins	↑DOX sensitivity and apoptosis towards proliferative cells	[116]
23	TQ	MDA-MB-231	↓Beclin-1, ↓VEGF, ↓Integrin-β1, ↓MMP-2,9	↓proliferation and migration, ↓Autophagy, ↓colony formation	[117]
24	TQ	DU-145, PC-3, LNCaP	↓p-Akt, ↓NF-κB↓MMP-3, ↓MMP-7	↓IL-7-induced tumor progression and metastatic invasion in PC-3 cells	[118]
25	TQ (50, 100 μM)	MCF-7, HepG2	↓sphingosine-1-phosphate (S1P), ↓ceramide-1-phosphate (C1P), ↓NF-κB1 mRNA, ↓NF-κB, ↓p65 protein levels, ↑neutral sphingomyelinase (N-SMase) enzyme activity, ↑cellular levels of C16-C24 ceramides and ↑cleaved caspase-3; ↑glucose-regulated protein 78-kd (GRP78) mRNA and protein	↑ceramide accumulation and ER stress in conjunction with ↓S1P, C1P, and NF-κB mediated cell survival ↑cancer cell death by triggering apoptosis	[119]
26	TQ (10 mM) + Difluoromethylornithine (0.5 mM)	T lymphoblastic leukemia (ALL) Jurkat cell line	↓UHRF1, ↓DNMT1, ↓HDAC1	Synergism, ↓cancer cell viability and ↑apoptosis	[120]
27	TQ and Cur	NLF, NB69, SK-N-BE(2)		↓proliferation, ↑apoptosis	[121]
28	TQ (50–100 µM) + FA (450 µM)	MDA-MB 231	↓PI_3_K/Akt pathway	Synergism in ↓cancer cell proliferation	[122]
29	TQ (20–100 μM)	C6 glioma cells	↑H_2_O_2_ generation, ↑microconidial ROS, ↓intracellular GSH level, ↓NF-κB, ↓PI3K, and AKT activation	↑apoptosis, ↓proliferation, and ↓glioma cell viability	[123]
30	TQ (20–60 μM)	786-O, 786-O-SI3, BFTC-909	↓Nanog, ↓Nestin, ↓Bid, ↑RO ↓CD44, ↓Oct-4, ↓Bcl-2, ↑cytochrome c, ↓phosphorylation of mTOR (Ser2448 and 2481) and AKT (Ser473)	↓the proliferation of renal cell carcinoma cells via ROS-induced apoptosis	[124]
31	TQ (0.5 μM)	HeLa cells	↓ROS generation	↓cancer cells proliferation	[125]
32	TQ (1–30 μM)	A431 cells	↑intracellular ROS, ↑p53, ↑Bax, ↓Mdm2, ↓Bcl-2, ↓Bcl-xl, ↓STAT3, ↑caspase-9,7 and 3; ↓phosphorylation of the upstream kinase, ↓Src, ↓cyclin D1, ↓survivin	↑apoptosis, ↓cell viability in dose-dependent manner	[126]
33	TQ (20 μmol/L TQ)	LoVo	↑p-PI3K, ↓p-Akt, ↓p-GSK3β, ↓β-catenin, ↓COX-2 expression; ↓PGE2 levels and the suppression of EP2 and EP4 activation	↓cancer cell proliferation. ↓cell migration	[127]
34	TQ (5 μM)	A549	↑Bax and ↓Bcl2 and ↑Bax/Bcl2 ratio, ↓cyclin D and ↑p21, ↑TRAIL receptor 1 and 2, ↓NFκB, ↓IKK1	↑G2/M cell cycle arrest, ↑apoptosis	[128]
35	TQ + DTX	DU145, C4-2B	↓PI3K/AKT, ↑BAX and ↑BID, ↑caspase-3, ↑PARP and ↓BCL-XL	↑cytotoxicity and ↑apoptosis	[129]
36	TQ (10–40 μM) +Dox (50–100 nM)	HTLV-1 positive (HuT-102) and HTLV-1 negative (Jurkat) CD4+ malignant T-cell lines	↑ROS, ↓tumor volume, ↓MMP	↓cell viability, induced apoptosis	[130]
37	TQ (2 μM,)	Irinotecan-resistant (CPT-11-R) LoVo colon cancer cells	Activate JNK and P38 and MOMP	↑the total cell death index and ↑apoptosis	[131]
38	TQ (2–100 µM)	A431 and Hep2	↑Bax/Bcl-2 ratio, ↓Akt and JNK phosphorylations	↓tumor volume and mass; ↑apoptosis; ↓cell proliferation	[132]
39	TQ (10–60 mM)	B16-F10	↓p-STAT3, p-JAK2 expression, and p-STAT3, ↑Bax and ↑caspase-3, ↓VEGF-A, ↓MCP-1, ↓TGF-b1, ↓RANTES, and↓IL-1β	↑cytotoxicity; ↑apoptosis	[133]
40	TQ (10 mM)	A549	↑Bax/Bcl-2, ↑p53; ↑caspases-3 and 9	↓cells viability; ↑apoptosis	[134]
41	5-FU + TQ	HCT116	↓WNT/ß-Catenin and PI3K/AKT, ß-Catenin	↓angiogenesis	[135]
42	TQ (10 mg/kg)	MDA-MB-231	↑E-cadherin mRNA expression	↓proliferation, migration, ↓invasion of cancer cells.	[136]
43	TQ (36 μg/mL) + tylophorine (88 μg/mL)	Hela cells		↑cell arrest in the G2/M phase	[137]
44	TQ (20 µM)	Jurkat cells, MDAMB-468 cells	↓UHRF1), ↓DNMT1 G9A, ↓HDAC, DNA methylation and histone post-translational modifications	↑tumor suppressor genes	[64]
45	TQ (40 µM)	A498	↑Bax, ↓Bcl-2, ↓Akt phosphorylation	↓proliferative, ↑apoptosis	[138]
46	TQ (1–10 µM)	HEK293 cells, Caki-1, A498	↓HIF-1α-mediated glycolysis via ubiquitination-proteasome dependent pathway	↓cancer cell angiogenesis	[139]
47	TQ (10–100 µM)	HeLa cells (Cancer)		↓dose-dependent cellular viability	[140]
48	TQ (0.5 mM) + cyclophosphamide (20 µM)	Her2+SKBR-3 and Her2- MDA-231	↓PI3K/Akt signaling, ↑PTEN, ↓cyclin D	synergistic cells death	[141]
49	TQ (0.003 mg/mL)	HSC-3, HSC-4, oral fibroblast, HACAT cell line		Dose and time-dependent cytotoxicity	[142]
50	TQ (0–80 µM)	PC3 cell line	↑ROS, ↓MCL-1, ↓MCL-XL, ↑BAX, ↑AIF, ↑cytochrome c	induced apoptosis	[143]
51	TQ	AGS(CRL-1739) cell line	VEGF-A gene expression	induced apoptosis	[144]
52	TQ	KB cells	↓activation of PI3K/Akt pathway.	↓proliferation, ↓migration, and invasion	[145]
53	TQ (60 μmol/L)	786-O, ACHN	↑p-AMPK w, ↓p-mTOR, ↑p-S6K	↓metastasis, induce autophagy	[146]
54	TQ+ gemcitabine	MCF-7, T47D	↓CD44^+^/CD24^−^ cell clone	Potentiate gemcitabine efficacy	[147]
55	TQ (0.5–20 µM)	769-P and 786-O	↑E-cadherin, ↓Snail, ↓ZEB1 expression, ↑LKB1 phosphorylation, ↑AMPK	↓metastasis	[148]
56	TQ (40–80 µM)	T24 and 253J bladder cancer cell	↓Bcl-2, ↓Bcl-xl, ↑Bax, ↑release of cytochrome C and AIF, ↑cleaved subunits of caspase-3, 8, 7, and PARP	Induce proliferation and apoptosis	[149]
57	TQ (20–80 µM)	U87MG, U118MG, and A172	↑Par-4, ↑p53, ↑p21, ↑Rb, ↓lamin B1, ↓cyclin E, ↓cyclin-dependent kinase-2 (CDK-2)	↓Glioblastoma	[150]
58	Temozolomide (100 μM) + TQ (50 μM)	U87MG cell line.	↓MMP 2, ↓MMP-9	↑cytotoxicity, ↓cells invasion	[151]
59	TQ (1–30 μM)	Jurkat, HL60 and HeLa cell line	↑UHRF1 degradation, ↑cleaved caspase-3 and ↑p73	↑apoptosis	[152]
60	TQ (10 mg/kg 5–200 µM)	B16, F10	↓p-STAT3, ↑DNA damage, and ↑ intracellular ROS	↑apoptosis	[153]
61	TQ (20, 100 mg/kg) IV	MDA-MB-231, MDA-MB-436,	↓elongation factor 2 kinase, ↓Src/FAK, ↓Akt, ↑miR-603, ↓NF-kB	↓tumor growth	[98]

Abbreviations: MMP—matrix metalloproteinase; STAT3—Signal transducer and activator of transcription-3; PTEN—Phosphatase and tensin homolog; eEF-2K—Eukaryotic elongation factor-2 kinase; NLRP3—NACHT, LRR, and pyrin domain-containing protein 3; ROS—reactive oxygen species; DOX—doxorubicin; SOD—Superoxide dismutase; LC-3—light chain 3-II; PPAR-γ—Peroxisome proliferator-activated receptor gamma; Ubiquitin-like; containing PHD and RING finger domains-1; p-mTOR—phosphorylated mechanistic target of rapamycin; NFκB—Nuclear factor kappa-light-chain-enhancer of activated B cells; 4E-BP1—Eukaryotic translation initiation factor 4E-binding protein 1; eIF4E—Eukaryotic translation initiation factor 4E; p70S6K—Ribosomal protein S6 kinase beta-1, also known as p70S6K kinase; FAK—focal adhesion kinase; FA—Ferulic Acid; Hes1—hairy and enhancer of split-1; VEGF—Vascular endothelial growth factor; IRAK1—Interleukin-1 receptor-associated kinase 1; TWIST1—Twist-related protein 1; DNMT1—DNA Methyltransferase 1; HDAC1—Histone deacetylase 1; Oct-4—octamer binding transcription factor-4; Nestin—Neuroepithelial stem cell protein; MDM2—Mouse double minute 2 homolog; p-GSK3β Glycogen synthase kinase 3 beta; IKK1—Inhibitor of nuclear factor kappa B; PRAP—prolactin receptor associated protein; MOMP—Mitochondrial outer membrane permeabilization; RANTES—Regulated upon Activation, Normal T Cell Expressed and Presumably Secreted; UHRF1—Ubiquitin-like, containing PHD and RING finger domains, 1; HDAC—Histone deacetylases; BAX—BCL2-Associated X; ZEB1—Zinc Finger E-Box Binding Homeobox 1; LKB1—liver kinase B1; AMPK—AMP-activated protein kinase; AIF—apoptosis-inducing factor; CDK-2—cyclin-dependent kinase-2.

**Table 4:** In vivo applications of thymoquinone in the treatment of cancer (↓: decrease, ↑: increase).

S.N	Drug and Dose	Animal Model	Molecular Target	Outcome	Ref.
1	TQ, DOX, and TQ+DOX	Wistar albino rats	↑apoptotic index, caspase 3, and HSP90 expressions in the DOX group	↓DOX toxicity	[154]
2	Cisplatin+ TQ+ vitamin E	Wistar rats	↓Catalase, ↓glutathione peroxidase, ↓SOD, and ↓reduced glutathione levels	↑cisplatin effect, ↓oxidative stress, ↓cisplatin toxicity	[155]
2	TQ (5–25 μM)	LPS/D-galactosamine induced acute hepatitis and HCl/EtOH-induced gastritis mouse model	↓(AP)-1/NF-κB pathways, ↓iNOS; ↓NO, ↓TNF-α; ↓COX-2, ↓IL-6, ↓PGE2, ↓IL-1β; ↓IRAK1	↓inflammatory response	[156,157]
3	TQ (1–25 µM)	Caki-1 cells, xenograft mouse model	↑p53; ↑Bax; ↓Bcl-2; ↓Bcl-xl, ↓cyclin D1, ↓cyclin D2, and ↓survivin via suppression of JAK2/STAT3 signaling pathway	Induces apoptosis via accumulation of ROS, ↓tumor growth	[84]
4	TQ (20 mg/kg) and pentoxifylline (15 mg/kg)	female albino mice	↓Notch1, ↓Hes1, ↓Jagged1, ↓β-catenin, ↓TNF-α, ↓IL-6, ↓IFN-γ, and ↓VEGF with ↑in IL-2, ↑CD4, ↑CD8, and↑apoptotic cells	↑chemotherapeutic effect of cisplatin by targeting Notch signaling pathway, ↓tumor growth	[158]
4	TQ 50 mg/kg	Colorectal cancer in SD rats	↑Antioxidant activity	Protective and preventive measure in cancer management	[159]
5	TQ (20 mg/kg)	SD rat	↑TRAIL/TRAILR2, ↑caspase-3, and ↓Bcl-2 downregulation, ↓TGF-β1 gene expression level. ↑hepatic GSH level and marked ↓hepatic MDA level, ↓alpha-fetoprotein level	↓HCC progression, ↑apoptosis	[160]
6	20 mg/kg BW	Diethylnitrosamine induced HCC in rats.	↓EGFR/ERK1/2 activation	protective effect against HCC	[161]
7	TQ	Hamster oral cancer Induced by DMBA	↓PI3K/AKT/mTOR signaling pathways ↓the mRNA expression level of NF-κBp50/p65	↑chemopreventive activity	[162]
8	TQ(5 mg), 6-MP (5 mg/kg)	Albino rats	↑spermatogenesis, ↓P53, ↓caspase-3 apoptotic pathway, ↑PI3K; ↓TNF-α	↓6-MP induced testicular damage, ↑its anticancer potential	[163]

Abbreviations: HSP90—heat shock protein 90; DOX—doxorubicin; SOD—Superoxide dismutase; COX—Cyclooxygenase; IRAK1—interleukin-1 receptor-associated kinase 1; STAT-3—Signal transducer and activator of transcription 3; NOTCH1—Notch homolog 1, translocation-associated (Drosophila); TRAIL—Tumor necrosis factor-related apoptosis-inducing ligand; ERK1—Extracellular signal-regulated kinase 1; HCC—hepatocellular carcinoma; TGF-β1—Transforming growth factor beta 1; PI3K—Phosphoinositides, including 3-kinase; 6-MP—6-mercaptopurine; DMBA—dimethylbenz(a)anthracene.

**Table 5:** TQ nanocarrier in the management of cancer and inflammation (↓: decrease, ↑: increase).

S.N	Formulations	Animal Model/Cell Line	Major Finding	Ref.
1	Core-shell NPs of mesoporous silica	SW1088, A172, HCN2	pH driven TQ release in tumor acidic environment ↑cell cycle arrest	[179]
2	Docetaxel (DTX) and TQ in borage oil-based nanoemulsion	MCF-7 MDA-MB-231	↑DTX anticancer potential; ↓dose, ↑apoptosis	[207]
3	TQ-loaded Soluplus-Solutol HS15 mixed micelles 2	SH-SY5Y	↑solubility (10-fold), ↑neuroblastoma cell migration	[185]
4	TQ- Chitosan NPs (12.5–200 µg/mL)	HepG2	↓cancer cells proliferation, ↑antimetastasis	[181]
5	TQ-loaded methoxy poly (ethylene glycol)-b-poly(“-caprolactone-NPs	MCF-7, PANC-1, Caco-2 Balb/c mice	↑oral BA (1.3-fold), ↑Solubility, ↑cancer cells selectivity	[183]
6	TQ loaded Soy phytosomes	A549	Improved release pattern; ↑the dose-dependent anticancer effect, ↑apoptotic induction	[184]
7	TQ-capped iron oxide NPs (TQ-IONPs)	MDA-MB-231	↑BA; ↑cellular uptake of TQ-IONPs; synergize the chemo-photothermal effect	[205]
8	TQ loaded radio-iodinated folic acid-chitosan NPs	SKOV-3 Caco-2	Folate receptor-mediated NPs ↑cellular internalization, ↑targeting to ovarian cancer cell	[195]
9	TQ loaded technetium-99m based NPs (^99m^Tc-TQ-NPs)	Rhabdo-myosarcoma cancer cells line	↑internalization and ↓externalization of radiopharmaceuticals; ↑anticancer potential	[203]
10	Cockle-shell-derived aragonite CaCl_3_ NPs for co-delivery of DOX and TQ	MBA MD231 3D	Co-delivery ↓cellular migration and invasion,	[186]
11	Glyceryl monooleate, cubosome for TQ delivery	MCF-7 MDA-MB-231	↑cytoplasmic accumulation; ↓cancer cells viability; ↑antitumor activity, ↑apoptosis	[187]
12	PLGA-PEG-Pluronic-TQ-NPs	Tamoxifen resistant breast cancer cells UACC 732, MCF-7	↑EE, sustained release, ↑targeted delivery, selective cytotoxicity to UACC 732	[174]
13	Vitamin-E-TPGS lipospheres for codelivery of cabazitaxel and TQ	MCF-7 MDA-MB-231	↑cellular internalization ↑anticancer potential,	[176]
14	Chitosan grafted lipidic nanocapsules for co-delivery of DTX and TQ	TNBC MCF-7	↑intracellular dual drug payload, escape endosomal effect, ↑anti-angiogenic effect, ↑cytotoxicity	[170]
15	Carum- and TQ loaded niosomes for target breast cancer cells	MCF-7, CaSki, SiHa	↑solubility, ↑BA and ↑permeability, ↓Cell Migration, ↑cytotoxicity	[10]
16	TQ and Cur loaded fluorescent liposomes	A549	↑cellular internalization ↓cellular proliferation, ↑cancer cells cytotoxicity	[204]
17	TQ loaded mesoporous silica NPs	HeLa MCF-7	↓effective dose (8-fold), ↑aqueous solubility, ↑cellular internalization ↓cell migration, ↑cytotoxicity, ↑apoptosis	[178]
18	TQ-NLC	HepG2 3T3	↑cellular accumulation driven by time and dose; modulate cellular morphology, ↑anticancer potential	[182]
19	TQ loaded SLN of phospholipon 90G	Carrageenan induced paw edema in rat	↑BA, ↑anti-inflammatory potential ↓paw edema, ↑antioxidant potential	[1]
20	Ethosomes for topical TQ delivery	Carrageenan rat paw edema	↑EE, ↑skin deposition ↓skin irritation	[51]
21	TQ loaded chitosan, pluronic F127 liposome for topical delivery	Carrageenan-induced paw edema	↑EE, ↑skin penetration ↑anti-inflammatory activity	[52]
22	SNEDDSs containing black seed oil and cur	Carrageenan-induced paw edema	↑entrapment efficiency, ↑transdermal penetration ↑anti-inflammatory activity	[208]
23	black seed oil loaded egg yolk liposomes	Eddy hot plate method in Swiss albino mice	↑BA; ↑EE, ↑anti-inflammatory activity	[209]
24	TQ and piperine loaded micro vehicle of guar gum	HepG2 cell lines	pH-responsive delivery ↓lethal dose ↑bactericidal activity ↓minimum inhibitory dose	[206]
25	Bio-SNEDDSs for co-delivery of cur and TQ	MCF-7 cells	↑drug loading, ↓cell viability	[210]
26	Fluorescent organic NPs	A549, HeLa SiHa, HEK-293T	↑BA, theranostic applications	[211]
27	TQ and resveratrol loaded silica NPs	HeLa cell line	↑EE, ↑drug loading, ↑apoptosis	[212]
28	chitosan-based nanocarrier for the encapsulation of NS oil	HCT 116 (colorectal carcinoma), PC3 (prostatic cancer)	dose-dependent ↓cell viability	[213]
29	TQ Pluronic NPs	MCF7 cells	↑TQ encapsulation, ↑cytotoxicity	[214]
30	TQ-NP of polystyrene-block-poly(ethylene oxide) diblock polymer	MCF-10-A cells MCF-7 cells, MDA-MB-231 cells	↑cellular uptake; ↑cytotoxicity	[215]
31	pH-sensitive multilamellar gold niosomes along with Akt-siRNA	tamoxifen-resistant T-47D and Akt-overexpressing MCF-7 cells	↑TQ delivery at cancer cell; ↑anticancer potential, resensitized T-47D cells	[200]
32	polysaccharide microcontainers of chitosan, xanthan gum soybean oil, and Nile red for TQ delivery	mouse melanoma M-3 cell	↑cellular uptake, ↓nonspecific toxicity; ↑antitumor effect	[216]
33	Myristic acid-chitosan nanogels	MCF-7	↑solubility, ↑cellular uptake	[217]
34	ketoprofen and TQ loaded mesoporous core-shell silica spheres	MDN- and XG-2-type myeloma cancer cells lines (IL-6 dependent)	↑cellular uptake and accumulation, ↑apoptosis	[218]
35	TQ loaded (PLGA)-NPs	MDA-MB-231	↑EE, ↑cancer cells toxicity	[219]
36	TQ loaded silver NPs	MDA-MB-231	↑cancer cells radiosensitivity	[220]

Abbreviations: NS—*Nigella sativa*; EE—entrapment efficiency; DTX—docetaxel; DOX—doxorubicin; TQNPs—thymoquinone nanoparticle; PLGA—poly(lactic-co-glycolic acid); Cur—curcumin; SNEDDS—self-nanoemulsifying drug delivery systems.

**Table 6:** Surface-modified TQ nanocarrier in the management of cancer (↓: decrease, ↑: increase).

S.N	Formulations	Animal Model/Cell Line	Major Finding	Ref.
1	Chitosan- (CS)-coated poly(d,l-lactide-co-glycolide) NPs	MDA-MB-231 MCF-7	↑Intestinal permeation; ↑BA; ↓dose and dosing frequency, ↑antioxidant potential	[180]
2	Anisamide coated TQ loaded lipidic core nanocapsules shell of eudragit S100	HT-29, HCT-116, Caco-2	Anisamide coating ↑colonic delivery of TQ due to specific binding with overexpressed sigma receptor	[192]
3	RNA aptamer A10 coated TQ loaded planetary ball-milled NPs of starch PCL, and PEG for specific bindings to prostate-specific membrane antigen overexpressed ABC transporter genes	DOX resistant C4-2B-R and LNCaP-R cells with a high expression of Hh	↑targeted delivery ↑circulations time, resensitized cancer cells for DOX	[193]
4	TQ loaded porous PVPylated Fe_3_O4 nanostructures	MDA-MB-231	↑ROS related cell death, ↑water-solubility, pH-dependent cellular delivery, ↑apoptosis	[201]
5	TQ loaded hyaluronic acid-decorated Pluronic^®^ NPs	MDA-MB-231, MDA-MB-468), murine (4T1), chick embryos	↓cell migration at a low dose; ↑circulation time; ↑cancer cells targeting	[194]
6	PEGylated vitamin-E TPGS-lipidic nanocapsules for co-delivery of DTX and TQ	MCF-7 and MDA-MB-231	PEGylation ↑circulation time; Re-sensitized the resistant TNBC cells; ↓side effects; ↑anti-metastatic effects	[173]
7	Chitosan coated PLGA-NPs for TQ delivery	A375	↑cellular accumulation; sustained delivery ↑cytotoxicity,	[188]
8	Poly-L-lysine and PEG-coated polysaccharide nanocontainers of diethylaminoethyl dextran/xanthan gum for TQ delivery	MCF-7 cells	↑cellular accumulation ↑cytotoxicity	[177]
9	Eudragit L-100 chitosan, HPMC, and PVA NPs of TQ for colon cancer treatment	Caco-2	↑colonic drug delivery ↑cytotoxicity	[202]
10	PEGylated liposome of dihexadecanoyl-sn-glycero-3-phosphocholine for co-delivery of DTX and TQ	MCF-7	↑drug encapsulation ↓docetaxel dose, ↑cancer cells cytotoxicity	[171]
11	Transferrin decorated TQ loaded PEG-PLGA-NPs		↑cellular accumulation ↓therapeutic dose ↓onset time, ↑cytotoxicity	[196]
12	AS1411-conjugated nanodroplets of phospholipids 1,2-dipalmitoyl-sn-glycero-3-phosphocholine	MDA-MB-231	Specific binding with overexpressed nucleolin on to cancer cell surface, ↑cytotoxic potential	[197]
13	PEGylated LMW TQ-loaded chitosan nanocapsules	MCF 7, HEK 293	↑absorption, ↑BA ↑cancer cells targeting	[175]

Abbreviations: BA—bioavailability; CS—chitosan; PEG—polyethylene glycol; LMW—low molecular weight; PLGA—poly(lactic-co-glycolic acid); PVA—polyvinyl alcohol; HPMC—hydroxypropyl methylcellulose; TPGS—D-α-tocopheryl polyethylene glycol succinate; DTX—docetaxel; PCL—polycaprolactone; ROS—reactive oxygen species.

**Table 7:** Patents of thymoquinone (TQ) and their nanocarrier systems related to inflammation and cancer (↓: decrease, ↑: increase).

S.N	Patent no	Type of Formulations	Product Claim and Activity	Outcome	Reference
1	WO2016024145A1WIPO (PCT)	TQ derivative	Cancer treatment	↑Anticancer effects	[230]
2	WO2018134852A1WIPO (PCT)	Vesicular formulations	Treatment of dermal inflammatory disorders	↑Bioavailability	[231]
3	WO2013030669A4WIPO (PCT)	TQ, TQ + eicosapentaenoic acid	Inflammation management including eicosapentaenoic acid	↓Inflammatory symptoms	[232]
4	WO2016167730A1WIPO (PCT)	Nanomicelles	Nanomicelles loaded with drug and H5WYG peptides for anticancer activity	↑Targeted delivery for cancer cells	[227]
5	US20160101124A1	Nanoliposome loaded with TQ and aminoglycoside	Nano-liposomal aminoglycoside-TQ formulations for administration to the mammal	↑bactericidal activity, ↓renal toxicity	[229]
6	WO-2016005786-A1	The liposome of TQ and taxane,	Liposomal formulations comprising TQ and taxane, and methods of treating cancer using the same	Synergize anticancer effect, ↑capsulation efficiency of the taxane ↑liposomes stability	[233]
7	CN-110420203-A	TQ	Application of the TQ in preparation prevention intravascular stent restenosis medicaments	↓intravascular diseases such as in-stent restenosis	[234]
8	US10485837B2	black cumin extract.	NS seeds component for management of anxiety, stress, and sleep disorders	Improve cognitive function	[235]
9	WO-2011126544-A2	TQ+ gemcitabine/oxaliplatin,	TQ analogs for the treatment of pancreatic cancer	↓drug resistance, ↑chemotherapeutic activity against pancreatic cancer	[236]
10	US-6218434-B1	TQ and dithymoquinone	Use of the naturally occurring quinones TQ and dithymoquinone as antineoplastic and cytotoxic agents	↓drug sensitivity against multi-drug resistant human cancers	[237]
11	CN-103288618-A	TQ synthesis method	A synthesis method of TQ serving as blood vessel inhibition medicament	A Synthesis method of TQ serving for blood vessel inhibition drug	[238]
12	CN-103833871-A	Hyaluronic acid-adipodihydrazide-TQ-grafted polymer	TQ grafted polymer for tumors specific delivery	↑tumors targeting, pH-dependent drug release	[239]
13	US-8029831-B2	TQ containing NS seed extract + cranberry fruit extract/	Management of microbial infections of the female urinary tract.	↓Urine pH, ↑antimicrobial activity, ↓inflammation and pain, ↓physiological stress.	[240]
14	DE-19844022-C1	Iron-binding glyco proteins (lactoferrin) and/or 10-hydroxy- 2-decenoic acid + TQ	use of iron-binding glycoproteins and/or 10-hydroxy-2-decenoic acid in combination with TQ for treatment of AIDS and other immunodeficiency diseases.	↓HIV plaques	[241]
15	US20190192686A1	Nanodroplet micelle	Cancer management	↑targeted delivery of anticancer drugs, ↓systemic toxicity.	[228]

**Table 8:** Some recent thymoquinone clinical trials.

S.N	Clinical Trial ID	Title	Trial Status	Age and Patient Inclusion Criteria	Intervention	Conditions	Sponsor	Target Size	Source
1	NCT03208790	Clinical and immunohistochemical evaluation of the cancer chemopreventive effect of thymoquinone compared to placebo on oral potentially malignant lesions among an Egyptian population: a randomized clinical trial	Phase 2	18–25 years Patients with any known potentially malignant lesion confirmed histologically	100 mg 200 mg Placebo oral capsule	Premalignant Lesion	Cairo University, Egypt	81	https://clinicaltrials.gov/show/NCT03208790; accessed on 12 January 2021
2	IRCT2016100914106N5	Preparation of oral gel-made from thymoquinone (TQ), and a clinical study investigating the efficacy of it on patients with aphtha	2	Patient possessing aphthous ulcer		Recurrent Aphthous Stomatitis	Kermanshah University of Medical Science, Iran	56	http://en.irct.ir/trial/13800; accessed on 12 January 2021
3	IRCT2016021826637N1	Evaluation effect of mucoadhesive NS in the treatment of chronic periodontitis	2	Patients who had not undertaken periodontal therapy in the past 3 months	Mucoadhesive Locally Delivery NS extract 0.2% and Thymoquinone 0.02%.	Chronic periodontitis	The ethics committee of Kermanshah University of Medical Science, Iran	20	http://en.irct.ir/trial/22014; accessed on 12 January 2021
4	NCT03776448	The effect of 2 g daily supplementation of thymoquinone -containing sativa nigra oil on blood glucose levels of adults: a placebo-controlled double-blinded randomized controlled trial	N/A	18–60 years of regular Student or Faculty in Sulaiman Al Rajhi Colleges	18–60 years	Diabetes mellitus	Sulaiman Al Rajhi Colleges, Saudi Arabia	30	https://clinicaltrials.gov/show/NCT03776448; accessed on 12 January 2021
5	CTRI/2018/11/016334	A randomized, open-label, prospective, three-arm, parallel, multicenter study to evaluate efficacy and safety of metformin with/without concomitant administration of thymoquinone in patients with type 2 diabetes mellitus.	2	Patients aged 18–65 years with type 2 diabetes mellitus and (BMI) between 18–30 kg per meter square		Type 2 diabetes mellitus without complications	Intas Pharmaceuticals Ltd., India	60	http://www.ctri.nic.in/Clinicaltrials/pmaindet2.php?trialid=28562; accessed on 12 January 2021
6	CTRI/2020/05/025167	Evaluation of efficacy and safety of thymoquinone compared to best supportive care in patients with covid-19	Phase 2	Confirmed COVID-19 patient (either sex) aged 18–65 years	50 mg tablet for 14 days as an add-on to best supportive as per guidelines of clinical management of COVID-19 as issued by MOHFW	RR < 20, HR < 90, oxygen saturation (pulse oximetry) >93% on room air at screening	Intas Pharmaceuticals Ltd., India	100	http://ctri.nic.in/Clinicaltrials/showallp.php?mid1=43378&EncHid=&userName=thymoquinone; accessed on 12 January 2021
7	NCT04476420	Comparison of NS oil with conventional management on clinical outcomes in oral submucous fibrosis	Phase 3	18 years clinically diagnosed patients (either sex) of oral submucous fibrosis	Topical application of N. sativa seed oil over buccal mucosa (1 mL) three times a day for 10 min (3–5 min on each side)	Oral submucous fibrosis	Ziauddin University, Pakistan	40	https://clinicaltrials.gov/ct2/show/NCT04476420; accessed on 12 January 2021
8	NC T04292314	Impact of combination therapy between hydroxyurea, omega 3, NS, and honey on antioxidant-oxidant status and reduction of iron overload in pediatric major thalassemia	Phase 3	Any case with the full manifestation of β-Thalassemia major disease Aged from 7–15 years old	1 g black seed oil contains 1% thymoquinone per day for 8 consecutive months up to 10 months		Beni-Suef University (Egypt)Maternity and Children Hospital, Makkah University of Arizona (Saudi Arabia)	350	https://clinicaltrials.gov/ct2/show/NCT04292314?cond=thymoquinone&draw=2&rank=3; accessed on 12 January 2021
9	CTRI/2020/12/029514	An open-label, balanced, randomized, three-treatment, single-period, single oral dose, parallel, exploratory pharmacokinetic study of thymoquinone tablet 12.5 mg, 25 mg, 50 mg in normal, healthy, adult, human subjects under fasting condition	Not yet recruiting	18.00–45.00 year(s) A normal, healthy, adult having a Body Mass Index between 18.5 to 30.0	Dose—12.5 mg; 25 mg; 50 mg Frequency—a single oral dose; Dosage form— tablet; Route of administration—oral	Not having any significant diseases	Intas Pharmaceuticals Limited, Corporate House, Ahmedabad—380054, Gujarat, India	12	http://ctri.nic.in/Clinicaltrials/pmaindet2.php?trialid=49799&EncHid=&userName=thymoquinone; accessed on 12 January 2021
10	NCT04686461	Effect of TQ extracted from NS in the treatment of arsenical keratosis	Not applicable	Age: 19–65 years Arsenical keratosis: Presence of moderate to severe keratosis (>5 mm) in both palms and soles	NS seeds extract containing ointment dose—twice daily for 12 weeks	The patient did not receive a topical application of any drug for the last three months.Drinking arsenic-contaminated water (>50 µg/L) for at least more than 6 months	Bangabandhu Sheikh Mujib Medical University, Dhaka, Bangladesh	34	https://clinicaltrials.gov/ct2/show/NCT04686461?cond=NCT04686461&draw=2&rank=1; accessed on 12 January 2021

NS—*Nigella sativa*; TQ—thymoquinone.

## Data Availability

Not applicable.

## Abbreviation

TQ	Thymoquinone
NS	Nigella sativa
RA	Rheumatoid Arthritis
MTX	Methotrexate
MDA	Malondialdehyde
BA	Bioavailability (BA)
MAPK	Mitogen-activated protein kinase
LPS	Lipopolysaccharides
DTX	Docetaxel
CBZ	Cabazitaxel
HA	Hyaluronic acid
PVP	Polyvinylpyrrolidone
DMARDs	Disease-modifying antirheumatic drugs
PGE2	Prostaglandin E2
GSSG	Glutathione
MMP	Matrix metalloproteinase
RANKL	Receptor-activated nuclear factor kappa-B ligand
COX	Cyclooxygenase
ROS	Reactive oxygen species
STAT3	Signal transducer and activator of transcription-3
PARP	Poly-(ADP-ribose) polymerase
Mdm2	Murine double minute-2
MOMP	Mitochondrial outer membrane permeability
TQ-NPs	Thymoquinone Nanoparticles
NADPH	Nicotinamide adenine dinucleotide phosphate oxidase
IL	Interleukin
TNF-α	Tumor necrosis factor alpha
TLR	Toll-like receptors
NF-κB	Nuclear factor kappa light chain enhancer of activated B cells
NLRP3	NOD-like receptor family pyrin domain containing 3
FCA	Freund’s complete adjuvant
PLC1	Polo-like kinase 1
PI3K	Phosphoinositides, including 3-kinase
JAK/STAT	Janus kinase signal transducers and transcription
Fe-NTA	Fe (III) nitrilotriacetic acid
miRNA	microRNA
lncRNA	Long non-coding RNAs
PTEN	Phosphatase and tensin homolog
Eca109 cells	Esophageal cancer cells
miR-34a	MicroRNA-34a
MBC	Metastatic breast cancers
DTX	Docetaxel
PEG	Polyethylene
TNBC	Triple negative breast cancer
PLL	poly-L-lysine
PVP	Polyvinylpyrrolidone
NFATc1	Nuclear factor of activated T-cells, cytoplasmic 1
ROS	Reactive oxygen species
fMLF	N-Formylmethionine-leucyl-phenylalanine
eEF-2K;	Eukaryotic elongation factor-2 kinase
NLRP3	NACHT, LRR, and pyrin domain-containing protein 3
DOX	Doxorubicin
SOD	Superoxide dismutase
LC-3	Light chain 3-II
PPAR-γ	Peroxisome proliferator-activated receptor gamma
UHRF1	Ubiquitin-like, containing PHD and RING finger domains-1
p-mTOR	Phosphorylated mechanistic target of rapamycin
NFκB	Nuclear factor kappa-light-chain-enhancer of activated B cells
4E-BP1	Eukaryotic translation initiation factor 4E-binding protein 1
eIF4E	Eukaryotic translation initiation factor 4E
p70S6K	Ribosomal protein S6 kinase beta-1 also known as p70S6K kinase
PI3K	Phosphoinositides, including 3-kinase
IRAK1	Interleukin-1 receptor-associated kinase 1
FAK	Focal adhesion kinase
Hes1	Hairy and enhancer of split-1
VEGF	Vascular endothelial growth factor
IRAK1	Interleukin-1 receptor-associated kinase 1,
TWIST1	Twist-related protein 1
DNMT1	DNA Methyltransferase 1,
HDAC1	Histone deacetylase 1
Oct-4	Octamer binding transcription factor-4
Nestin	Neuroepithelial stem cell protein
MDM2	Mouse double minute 2 homolog
p-GSK3β	Glycogen synthase kinase 3 beta
TRAIL	Tumor necrosis factor-related apoptosis-inducing ligand
IKK1	Inhibitor of nuclear factor kappa B
PRAP	Prolactin receptor associated protein
UHRF1	Ubiquitin-like, containing PHD and RING finger domains 1
HDAC	Histone deacetylases
BAX	BCL2 Associated X
ZEB1	Zinc Finger E-Box Binding Homeobox 1
LKB1	Liver kinase B1
AMPK	AMP-activated protein kinase
AIF	Apoptosis-inducing factor
ERK1/2	Extracellular signal-regulated protein kinase
CDK-2	Cyclin-dependent kinase-2;
HCC	Hepatocellular carcinoma
RANTES	Regulated upon activation normal T cell expressed and presumably secreted
RES	Reticuloendothelial system

## References

[1] Rathore C., Upadhyay N.K., Sharma A., Lal U.R., Raza K., Negi P. (2019). Phospholipid nanoformulation of thymoquinone with enhanced bioavailability: Development, characterization and anti-inflammatory activity. J. Drug Deliv. Sci. Technol..

[2] Wang X., Fang G., Yang Y., Pang Y. (2019). The newly discovered natural compounds against rheumatoid arthritis—An overview. Phytochem. Lett..

[3] Ardah M.T., Merghani M.M., Haque M.E. (2019). Thymoquinone prevents neurodegeneration against MPTP in vivo and modulates α-synuclein aggregation in vitro. Neurochem. Int..

[4] Khan M.A., Aldebasi Y.H., Alsuhaibani S.A., Alsahli M.A., Alzohairy M.A., Khan A., Younus H. (2018). Therapeutic potential of thymoquinone liposomes against the systemic infection of Candida albicans in diabetic mice. PLoS ONE.

[5] Ansari M.O., Parveen N., Ahmad F., Wani A.L., Afrin S., Rahman Y., Jameel S., Khan Y.A., Siddique H.R., Tabish M. (2019). Evaluation of DNA interaction, genotoxicity and oxidative stress induced by iron oxide nanoparticles both in vitro and in vivo: Attenuation by thymoquinone. Sci. Rep..

[6] Khan M.A., Afzal M. (2016). Chemical composition of Nigella sativa Linn: Part 2 Recent advances. Inflammopharmacology.

[7] Faisal R., Ahmad N., Fahad Y.S., Chiragh S. (2018). Anti-Arthritic Effect of Thymoquinone in Comparison with Methotrexate on Pristane Induced Arthritis in Female Sprague Dawley Rats. J. Ayub Med. Coll. Abbottabad.

[8] Ahmad A., Mishra R.K., Vyawahare A., Kumar A., Rehman M.U., Qamar W., Khan A.Q., Khan R. (2019). Thymoquinone (2-Isopropyl-5-methyl-1, 4-benzoquinone) as a chemopreventive/anticancer agent: Chemistry and biological effects. Saudi Pharm. J..

[9] Bamosa A.O., Kaatabi H., Lebdaa F.M., Al Elq A.-M., Al-Sultanb A. (2011). Effect of Nigella sativa seeds on the glycemic control of patients with type 2 diabetes mellitus. Indian J. Physiol. Pharmacol..

[10] Barani M., Mirzaei M., Torkzadeh-Mahani M., Adeli-Sardou M. (2019). Evaluation of Carum-loaded Niosomes on Breast Cancer Cells:Physicochemical Properties, In Vitro Cytotoxicity, Flow Cytometric, DNA Fragmentation and Cell Migration Assay. Sci. Rep..

[11] Alkharfy K.M., Ahmad A., Khan R.M.A., Al-Shagha W.M. (2014). Pharmacokinetic plasma behaviors of intravenous and oral bioavailability of thymoquinone in a rabbit model. Eur. J. Drug Metab. Pharmacokinet..

[12] Rasheeda K., Samyuktha D., Fathima N.N. (2019). Self-association of type I collagen directed by thymoquinone through alteration of molecular forces. Int. J. Biol. Macromol..

[13] Nagi M.N., Almakki H.A. (2009). Thymoquinone supplementation induces quinone reductase and glutathione transferase in mice liver: Possible role in protection against chemical carcinogenesis and toxicity. Phytother. Res..

[14] Darakhshan S., Pour A.B., Colagar A.H., Sisakhtnezhad S. (2015). Thymoquinone and its therapeutic potentials. Pharmacol. Res..

[15] Mohammadabadi M., Mozafari M. (2018). Enhanced efficacy and bioavailability of thymoquinone using nanoliposomal dosage form. J. Drug Deliv. Sci. Technol..

[16] Khalife K.H., Lupidi G. (2007). Nonenzymatic reduction of thymoquinone in physiological conditions. Free Radic. Res..

[17] Islam M.T., Sultana N., Alam Riaz T., Ferdous J., Guha B., Mohagon S., Mutsuddy R., Santos J.V.D.O., Dos Reis A.C., Braga A.L. (2016). Thymoquinone is knocking at the door of clinical trial. Int. Arch. Med..

[18] Badary O.A., Taha R.A., El-Din A.M.G., Abdel-Wahab M.H. (2003). Thymoquinone is a Potent Superoxide Anion Scavenger. Drug Chem. Toxicol..

[19] Mahmoud Y.K., Abdelrazek H.M. (2019). Cancer: Thymoquinone antioxidant/pro-oxidant effect as potential anticancer remedy. Biomed. Pharmacother..

[20] Widdifield J. (2019). Preventing Rheumatoid Arthritis: A Global Challenge. Clin. Ther..

[21] Sigaux J., Biton J., André E., Semerano L., Boissier M.-C. (2019). Air pollution as a determinant of rheumatoid arthritis. Jt. Bone Spine.

[22] Cush J.J. (2021). Rheumatoid Arthritis. Med. Clin. N. Am..

[23] Boissier M.-C., Biton J., Semerano L., Decker P., Bessis N. (2020). Origins of rheumatoid arthritis. Jt. Bone Spine.

[24] Qamar T., Mukherjee S. (2021). Genetic approaches for the diagnosis and treatment of rheumatoid arthritis through personalized medicine. Gene Rep..

[25] Siouti E., Andreakos E. (2019). The many facets of macrophages in rheumatoid arthritis. Biochem. Pharmacol..

[26] Hajialilo M., Ghorbanihaghjo A., Maddahi S., Khabbazi A., Mahdavi A.M., Rashtchizadeh N. (2020). Association between serum Toll-like receptor 4 and 8-hydroxy-2′-deoxyguanosine levels with disease activity in rheumatoid arthritis patients. Egypt. Rheumatol..

[27] Tada Y., Koarada S., Morito F., Mitamura M., Inoue H., Suematsu R., Ohta A., Miyake K., Nagasawa K. (2008). Toll-like receptor homolog RP105 modulates the antigen-presenting cell function and regulates the development of collagen-induced arthritis. Arthritis Res. Ther..

[28] McInnes I.B., Schett G. (2017). Pathogenetic insights from the treatment of rheumatoid arthritis. Lancet.

[29] Chatzidionysiou K., Fragoulis G.E. (2019). Established rheumatoid arthritis—Redefining the concept. Best Pr. Res. Clin. Rheumatol..

[30] Hansildaar R., Vedder D., Baniaamam M., Tausche A.-K., Gerritsen M., Nurmohamed M.T. (2021). Cardiovascular risk in inflammatory arthritis: Rheumatoid arthritis and gout. Lancet Rheumatol..

[31] Jayashree S., Nirekshana K., Guha G., Bhakta-Guha D. (2018). Cancer chemotherapeutics in rheumatoid arthritis: A convoluted connection. Biomed. Pharmacother..

[32] Acar M., Sütçü M., Salman N., Somer A. (2017). The Risk of Tuberculosis and TNF-alpha Inhibitors. J. Pediatr. Infect..

[33] Law S.T., Taylor P.C. (2019). Role of biological agents in treatment of rheumatoid arthritis. Pharmacol. Res..

[34] Arjumand S., Shahzad M., Shabbir A., Yousaf M.Z. (2019). Thymoquinone attenuates rheumatoid arthritis by downregulating TLR2, TLR4, TNF-α, IL-1, and NFκB expression levels. Biomed. Pharmacother..

[35] Umar S., Zargan J., Umar K., Ahmad S., Katiyar C.K., Khan H.A. (2012). Modulation of the oxidative stress and inflammatory cytokine response by thymoquinone in the collagen induced arthritis in Wistar rats. Chem. Interact..

[36] Boudiaf K., Hurtado-Nedelec M., Belambri S.A., Marie J.-C., Derradji Y., Benboubetra M., El-Benna J., Dang P.M.-C. (2016). Thymoquinone strongly inhibits fMLF-induced neutrophil functions and exhibits anti-inflammatory properties in vivo. Biochem. Pharmacol..

[37] Umar S., Hedaya O., Singh A.K., Ahmed S. (2015). Thymoquinone inhibits TNF-α-induced inflammation and cell adhesion in rheumatoid arthritis synovial fibroblasts by ASK1 regulation. Toxicol. Appl. Pharmacol..

[38] Vaillancourt F., Silva P., Shi Q., Fahmi H., Fernandes J.C., Benderdour M. (2010). Elucidation of molecular mechanisms underlying the protective effects of thymoquinone against rheumatoid arthritis. J. Cell. Biochem..

[39] Thummuri D., Jeengar M.K., Shrivastava S., Nemani H., Ramavat R.N., Chaudhari P., Naidu V. (2015). Thymoquinone prevents RANKL-induced osteoclastogenesis activation and osteolysis in an in vivo model of inflammation by suppressing NF-KB and MAPK Signalling. Pharmacol. Res..

[40] Taka E., Mazzio E.A., Goodman C.B., Redmon N., Flores-Rozas H., Reams R.R., Darling-Reed S., Soliman K.F. (2015). Anti-inflammatory effects of thymoquinone in activated BV-2 microglial cells. J. Neuroimmunol..

[41] Chen W.-P., Tang J.-L., Bao J.-P., Wu L.-D. (2010). Thymoquinone inhibits matrix metalloproteinase expression in rabbit chondrocytes and cartilage in experimental osteoarthritis. Exp. Biol. Med..

[42] Tekeoglu I., Dogan A., Ediz L., Budancamanak M., Demirel A. (2007). Effects of thymoquinone (volatile oil of black cumin) on rheumatoid arthritis in rat models. Phytother. Res..

[43] Pop R.M., Sabin O., Suciu Ș., Vesa S.C., Socaci S.A., Chedea V.S., Bocsan I.C., Buzoianu A.D. (2020). Nigella Sativa’s Anti-Inflammatory and Antioxidative Effects in Experimental Inflammation. Antioxidants.

[44] van de Veerdonk F.L., Netea M.G., Dinarello C.A., Joosten L.A. (2011). Inflammasome activation and IL-1β and IL-18 processing during infection. Trends Immunol..

[45] Ciążyńska M., Bednarski I.A., Narbutt J., Lesiak A. (2020). NLRP1 and NLRP3 inflammasomes as a new approach to skin carcinogenesis (Review). Oncol. Lett..

[46] Bordoni L., Fedeli D., Nasuti C., Maggi F., Papa F., Wabitsch M., De Caterina R., Gabbianelli R. (2019). Antioxidant and Anti-Inflammatory Properties of Nigella sativa Oil in Human Pre-Adipocytes. Antioxidants.

[47] Xiao S., Tang Y., Lv Z., Lin Y., Chen L. (2019). Nanomedicine—Advantages for their use in rheumatoid arthritis theranostics. J. Control. Release.

[48] Pham C.T.N. (2011). Nanotherapeutic approaches for the treatment of rheumatoid arthritis. Wiley Interdiscip. Rev. Nanomed. Nanobiotechnol..

[49] Fang Z., Pan S., Gao P., Sheng H., Li L., Shi L., Zhang Y., Cai X. (2020). Stimuli-responsive charge-reversal nano drug delivery system: The promising targeted carriers for tumor therapy. Int. J. Pharm..

[50] García M.C., Makhlouf A.S.H., Abu-Thabit N.Y. (2019). 13-Stimuli-responsive polymersomes for drug delivery applications. Stimuli Responsive Polymeric Nanocarriers for Drug Delivery Applications.

[51] Kausar H., Mujeeb M., Ahad A., Moolakkadath T., Aqil M., Ahmad A., Akhter H. (2019). Optimization of ethosomes for topical thymoquinone delivery for the treatment of skin acne. J. Drug Deliv. Sci. Technol..

[52] Mostafa M., Alaaeldin E., Aly U.F., Sarhan H.A. (2018). Optimization and Characterization of Thymoquinone-Loaded Liposomes with Enhanced Topical Anti-inflammatory Activity. AAPS PharmSciTech.

[53] Dzaye O., Bødtker H., Reiter-Brennan C., Blaha M.J., Mortensen M.B. (2020). Danish National Trends in Cardiovascular Disease and Cancer Drug Expenditure in Relation to Trends in Cardiovascular Disease and Cancer Deaths. Am. J. Med..

[54] Hausman D.M. (2019). What Is Cancer?. Perspect. Biol. Med..

[55] Dawson M.A., Kouzarides T. (2012). Cancer Epigenetics: From Mechanism to Therapy. Cell.

[56] Mahaur S., Upadhyay S., Pal R.R. (2020). Indolizine: In-silico identification of inhibitors against mutated BCR-ABL protein of chronic myeloid leukemia. Res. J. Pharmacol. Pharmacodyn..

[57] Graham T.A., Sottoriva A. (2017). Measuring cancer evolution from the genome. J. Pathol..

[58] Khan A., Tania M., Fu S., Fu J. (2017). Thymoquinone, as an anticancer molecule: From basic research to clinical investigation. Oncotarget.

[59] Fardi M., Solali S., Hagh M.F. (2018). Epigenetic mechanisms as a new approach in cancer treatment: An updated review. Genes Dis..

[60] Groves M.D. (2003). The pathogenesis of neoplastic meningitis. Curr. Oncol. Rep..

[61] Khan A., Tania M., Fu J. (2019). Epigenetic role of thymoquinone: Impact on cellular mechanism and cancer therapeutics. Drug Discov. Today.

[62] Pang J., Shen N., Yan F., Zhao N., Dou L., Wu L.-C., Seiler C.L., Yu L., Yang K., Bachanova V. (2017). Thymoquinone exerts potent growth-suppressive activity on leukemia through DNA hypermethylation reversal in leukemia cells. Oncotarget.

[63] Imran M., Rauf A., Khan I.A., Shahbaz M., Qaisrani T.B., Fatmawati S., Abu-Izneid T., Imran A., Rahman K.U., Gondal T.A. (2018). Thymoquinone: A novel strategy to combat cancer: A review. Biomed. Pharmacother..

[64] Qadi S.A., Hassan M.A., Sheikh R.A., Baothman O.A., Zamzami M.A., Choudhry H., Al-Malki A.L., Albukhari A., Alhosin M. (2019). Thymoquinone-Induced Reactivation of Tumor Suppressor Genes in Cancer Cells Involves Epigenetic Mechanisms. Epigenetics Insights.

[65] Alobaedi O.H., Talib W.H., Basheti I.A. (2017). Antitumor effect of thymoquinone combined with resveratrol on mice transplanted with breast cancer. Asian Pac. J. Trop. Med..

[66] Rajput S., Kumar B.P., Dey K.K., Pal I., Parekh A., Mandal M. (2013). Molecular targeting of Akt by thymoquinone promotes G1 arrest through translation inhibition of cyclin D1 and induces apoptosis in breast cancer cells. Life Sci..

[67] Kou B., Liu W., Zhao W., Duan P., Yang Y., Yi Q., Guo F., Li J., Zhou J., Kou Q. (2017). Thymoquinone inhibits epithelial-mesenchymal transition in prostate cancer cells by negatively regulating the TGF-β/Smad2/3 signaling pathway. Oncol. Rep..

[68] Lei X., Lv X., Liu M., Yang Z., Ji M., Guo X., Dong W. (2012). Thymoquinone inhibits growth and augments 5-fluorouracil-induced apoptosis in gastric cancer cells both in vitro and in vivo. Biochem. Biophys. Res. Commun..

[69] Feng L.-M., Wang X.-F., Huang Q.-X. (2017). Thymoquinone induces cytotoxicity and reprogramming of EMT in gastric cancer cells by targeting PI3K/Akt/mTOR pathway. J. Biosci..

[70] Norwood A.A., Tan M., May M., Tucci M., Benghuzzi H. (2006). Comparison of potential chemotherapeutic agents, 5-fluoruracil, green tea, and thymoquinone on colon cancer cells. Biomed. Sci. Instrum..

[71] Zhang L., Bai Y., Yang Y. (2016). Thymoquinone chemosensitizes colon cancer cells through inhibition of NF-κB. Oncol. Lett..

[72] Kundu J., Chun K.-S., Aruoma O.I., Kundu J.K. (2014). Mechanistic perspectives on cancer chemoprevention/chemotherapeutic effects of thymoquinone. Mutat. Res./Fundam. Mol. Mech. Mutagen..

[73] Baillie K.E., Stirling P.C. (2021). Beyond Kinases: Targeting Replication Stress Proteins in Cancer Therapy. Trends Cancer.

[74] Bai T., Lian L.-H., Wu Y.-L., Wan Y., Nan J.-X. (2013). Thymoquinone attenuates liver fibrosis via PI3K and TLR4 signaling pathways in activated hepatic stellate cells. Int. Immunopharmacol..

[75] Dalli T., Beker M., Terzioglu-Usak S., Akbas F., Elibol B. (2018). Thymoquinone activates MAPK pathway in hippocampus of streptozotocin-treated rat model. Biomed. Pharmacother..

[76] Kandeil M.A., Mahmoud M.O., Abdel-Razik A.-R.H., Gomaa S.B. (2019). Thymoquinone and geraniol alleviate cisplatin-induced neurotoxicity in rats through downregulating the p38 MAPK/STAT-1 pathway and oxidative stress. Life Sci..

[77] Zhou Y., Jianhua C., Rehse P.H. (2010). Thymoquinone and Poloxin are slow-irreversible inhibitors to human Polo-like kinase 1 Polo-box domain. J. Med. Coll. PLA.

[78] Afrose S.S., Junaid M., Akter Y., Tania M., Zheng M., Khan M.A. (2020). Targeting kinases with thymoquinone: A molecular approach to cancer therapeutics. Drug Discov. Today.

[79] AbuKhader M. (2013). Thymoquinone in the clinical treatment of cancer: Fact or fiction?. Pharmacogn. Rev..

[80] Zidan A.-A.A., El-Ashmawy N.E., Khedr E.G., Ebeid E.-Z.M., Salem M.L., Mosalam E.M. (2018). Loading of doxorubicin and thymoquinone with F2 gel nanofibers improves the antitumor activity and ameliorates doxorubicin-associated nephrotoxicity. Life Sci..

[81] Fishbein A., Hammock B.D., Serhan C.N., Panigrahy D. (2021). Carcinogenesis: Failure of resolution of inflammation?. Pharmacol. Ther..

[82] Farkhondeh T., Samarghandian S., Hozeifi S., Azimi-Nezhad M. (2017). Therapeutic effects of thymoquinone for the treatment of central nervous system tumors: A review. Biomed. Pharmacother..

[83] Ma J., Zhang Y., Deng H., Liu Y., Lei X., He P., Dong W. (2020). Thymoquinone inhibits the proliferation and invasion of esophageal cancer cells by disrupting the AKT/GSK -3β/Wnt signaling pathway via PTEN upregulation. Phytother. Res..

[84] Chae I.G., Song N.-Y., Kim D.-H., Lee M.-Y., Park J.-M., Chun K.-S. (2020). Thymoquinone induces apoptosis of human renal carcinoma Caki-1 cells by inhibiting JAK2/STAT3 through pro-oxidant effect. Food Chem. Toxicol..

[85] Noel B., Singh S.K., Lillard J.W., Singh R. (2020). Role of natural compounds in preventing and treating breast cancer. Front. Biosci. (Sch. ed.).

[86] Pandey M.K., Gupta S.C., Nabavizadeh A., Aggarwal B.B. (2017). Regulation of cell signaling pathways by dietary agents for cancer prevention and treatment. Semin. Cancer Biol..

[87] Bimonte S., Albino V., Barbieri A., Tamma M.L., Nasto A., Palaia R., Molino C., Bianco P., Vitale A., Schiano R. (2019). Dissecting the roles of thymoquinone on the prevention and the treatment of hepatocellular carcinoma: An overview on the current state of knowledge. Infect. Agents Cancer.

[88] Talib W.H. (2017). Regressions of Breast Carcinoma Syngraft Following Treatment with Piperine in Combination with Thymoquinone. Sci. Pharm..

[89] Martucciello S., Masullo M., Cerulli A., Piacente S. (2020). Natural Products Targeting ER Stress, and the Functional Link to Mitochondria. Int. J. Mol. Sci..

[90] Veena M.S., Raychaudhuri S., Basak S.K., Venkatesan N., Kumar P., Biswas R., Chakrabarti R., Lu J., Su T., Gallagher-Jones M. (2020). Dysregulation of hsa-miR-34a and hsa-miR-449a leads to overexpression of PACS-1 and loss of DNA damage response (DDR) in cervical cancer. J. Biol. Chem..

[91] Valcourt D.M., Day E.S. (2020). Dual Regulation of miR-34a and Notch Signaling in Triple-Negative Breast Cancer by Antibody/miRNA Nanocarriers. Mol. Ther.-Nucleic Acids.

[92] Imani S., Wei C., Cheng J., Khan A., Fu S., Yang L., Tania M., Zhang X., Xiao X., Zhang X. (2017). MicroRNA-34a targets epithelial to mesenchymal transition-inducing transcription factors (EMT-TFs) and inhibits breast cancer cell migration and invasion. Oncotarget.

[93] Salem A.A., El Haty I.A., Abdou I.M., Mu Y. (2015). Interaction of human telomeric G-quadruplex DNA with thymoquinone: A possible mechanism for thymoquinone anticancer effect. Biochem. Biophys. Acta (BBA)-Gen. Subj..

[94] Zubair H., Khan H.Y., Sohail A., Azim S., Ullah M.F., Ahmad A., Sarkar F.H., Hadi S.M. (2013). Redox cycling of endogenous copper by thymoquinone leads to ROS-mediated DNA breakage and consequent cell death: Putative anticancer mechanism of antioxidants. Cell Death Dis..

[95] Karki N., Aggarwal S., Laine R.A., Greenway F., Losso J.N. (2020). Cytotoxicity of juglone and thymoquinone against pancreatic cancer cells. Chem. Interact..

[96] Barkat M.A., Pottoo F.H., Beg S., Rahman M., Ahmad F.J. (2020). Evidence-Based Review on Clinical Potential of Thymoquinone in Breast Cancer. Nanomed. Bioact..

[97] Guler E.M., Sisman B.H., Kocyigit A., Hatiboglu M.A. (2021). Investigation of cellular effects of thymoquinone on glioma cell. Toxicol. Rep..

[98] Kabil N., Bayraktar R., Kahraman N., Mokhlis H.A., Calin G.A., Lopez-Berestein G., Ozpolat B. (2018). Thymoquinone inhibits cell proliferation, migration, and invasion by regulating the elongation factor 2 kinase (eEF-2K) signaling axis in triple-negative breast cancer. Breast Cancer Res. Treat..

[99] Fröhlich T., Reiter C., Saeed M.E.M., Hutterer C., Hahn F., Leidenberger M., Friedrich O., Kappes B., Marschall M., Efferth T. (2018). Synthesis of Thymoquinone–Artemisinin Hybrids: New Potent Antileukemia, Antiviral, and Antimalarial Agents. ACS Med. Chem. Lett..

[100] Bhattacharjee M., Upadhyay P., Sarker S., Basu A., Das S., Ghosh A., Ghosh S., Adhikary A. (2020). Combinatorial therapy of Thymoquinone and Emodin synergistically enhances apoptosis, attenuates cell migration and reduces stemness efficiently in breast cancer. Biochem. Biophys. Acta (BBA)-Gen. Subj..

[101] Jehan S., Zhong C., Li G., Bakhtiar S.Z., Li D., Sui G. (2020). Thymoquinone Selectively Induces Hepatocellular Carcinoma Cell Apoptosis in Synergism With Clinical Therapeutics and Dependence of p53 Status. Front. Pharmacol..

[102] Ahmad I., Muneer K.M., Tamimi I.A., Chang M.E., Ata M.O., Yusuf N. (2013). Thymoquinone suppresses metastasis of melanoma cells by inhibition of NLRP3 inflammasome. Toxicol. Appl. Pharmacol..

[103] Ballout F., Monzer A., Fatfat M., El Ouweini H., Jaffa M.A., Abdel-Samad R., Darwiche N., Abou-Kheir W., Gali-Muhtasib H. (2020). Thymoquinone induces apoptosis and DNA damage in 5-Fluorouracil-resistant colorectal cancer stem/progenitor cells. Oncotarget.

[104] Bashir A.O., El-Mesery M.E., Anwer R., Eissa L.A. (2020). Thymoquinone potentiates miR-16 and miR-375 expressions in hepatocellular carcinoma. Life Sci..

[105] Kandeil M.A., Gomaa S.B., Mahmoud M.O. (2020). The effect of some natural antioxidants against cisplatin-induced neurotoxicity in rats: Behavioral testing. Heliyon.

[106] Vcherashniaya A.V., Martinovich I.V., Martinovich G.G., Shadyro O.I., Cherenkevich S.N. (2020). A Raman Spectroscopic Study of Thymoquinone Antitumor Action. J. Appl. Spectrosc..

[107] Racoma I.O., Meisen W.H., Wang Q.-E., Kaur B., Wani A.A. (2013). Thymoquinone Inhibits Autophagy and Induces Cathepsin-Mediated, Caspase-Independent Cell Death in Glioblastoma Cells. PLoS ONE.

[108] Lee H.J., Kim M.J., Kim Y.S., Choi M.Y., Cho G.J., Choi W.S. (2020). UHRF1 silences gelsolin to inhibit cell death in early stage cervical cancer. Biochem. Biophys. Res. Commun..

[109] Dera A.A., Rajagopalan P., Al Fayi M., Ahmed I., Chandramoorthy H.C. (2020). Indirubin-3-monoxime and thymoquinone exhibit synergistic efficacy as therapeutic combination in in-vitro and in-vivo models of Lung cancer. Arch. Pharm. Res..

[110] Alghamdi A.A., Mohammed M.R.S., Zamzami M.A., Al-Malki A.L., Qari M.H., Khan M.I., Choudhry H. (2020). Untargeted Metabolomics Identifies Key Metabolic Pathways Altered by Thymoquinone in Leukemic Cancer Cells. Nutrients.

[111] Ha J.H., Jayaraman M., Radhakrishnan R., Gomathinayagam R., Yan M., Song Y.S., Isidoro C., Dhanasekaran D.N. (2020). Differential effects of thymoquinone on lysophosphatidic acid-induced oncogenic pathways in ovarian cancer cells. J. Tradit. Complement. Med..

[112] Zhang M., Du H., Wang L., Yue Y., Zhang P., Huang Z., Lv W., Ma J., Shao Q., Ma M. (2020). Thymoquinone suppresses invasion and metastasis in bladder cancer cells by reversing EMT through the Wnt/β-catenin signaling pathway. Chem. Interact..

[113] Boyacioglu O. (2020). Interdependence of cytotoxic activities of bioactive components in black seed (*Nigella sativa* L.) essential oil and intracellular zinc levels. Fresenius Environ. Bull..

[114] Costa J., Keser V., Jackson C., Saraiva N., Guerreiro Í., Almeida N., Camões S., Manguinhas R., Castro M., Miranda J. (2020). A multiple endpoint approach reveals potential in vitro anticancer properties of thymoquinone in human renal carcinoma cells. Food Chem. Toxicol..

[115] Bashmail H.A., AlAmoudi A.A., Noorwali A., Hegazy G.A., Ajabnoor G.M., Al-Abd A.M. (2020). Thymoquinone Enhances Paclitaxel Anti-Breast Cancer Activity via Inhibiting Tumor-Associated Stem Cells Despite Apparent Mathematical Antagonism. Molecules.

[116] El-Far A.H., Darwish N.H.E., Mousa S.A. (2020). Senescent Colon and Breast Cancer Cells Induced by Doxorubicin Exhibit Enhanced Sensitivity to Curcumin, Caffeine, and Thymoquinone. Integr. Cancer Ther..

[117] Ünal T.D., Hamurcu Z., Delibaşı N., Çınar V., Güler A., Gökçe S., Nurdinov N., Ozpolat B. (2021). Thymoquinone Inhibits Proliferation and Migration of MDA-MB-231 Triple Negative Breast Cancer Cells by Suppressing Autophagy, Beclin-1 and LC3. Anti-Cancer Agents Med. Chem..

[118] Alshyarba M., Otifi H., Al Fayi M., A Dera A., Rajagopalan P. (2020). Thymoquinone inhibits IL-7-induced tumor progression and metastatic invasion in prostate cancer cells by attenuating matrix metalloproteinase activity and Akt/NF-κB signaling. Biotechnol. Appl. Biochem..

[119] Aslan M., Afşar E., Kırımlıoglu E., Çeker T., Yılmaz Ç. (2021). Antiproliferative Effects of Thymoquinone in MCF-7 Breast and HepG2 Liver Cancer Cells: Possible Role of Ceramide and ER Stress. Nutr. Cancer.

[120] Alhosin M., Razvi S.S.I., Sheikh R.A., Khan J.A., Zamzami M.A., Choudhry H. (2020). Thymoquinone and Difluoromethylornithine (DFMO) Synergistically Induce Apoptosis of Human Acute T Lymphoblastic Leukemia Jurkat Cells Through the Modulation of Epigenetic Pathways. Technol. Cancer Res. Treat..

[121] Korff J.M., Menke K., Schwermer M., Falke K., Schramm A., Längler A., Zuzak T.J. (2021). Antitumoral Effects of Curcumin (*Curcuma longa* L.) and Thymoquinone (*Nigella sativa* L.) on Neuroblastoma Cell Lines. Complement. Med. Res..

[122] Al-Mutairi A., Rahman A., Rao M.S. (2021). Low Doses of Thymoquinone and Ferulic Acid in Combination Effectively Inhibit Proliferation of Cultured MDA-MB 231 Breast Adenocarcinoma Cells. Nutr. Cancer.

[123] Krylova N.G., Drobysh M.S., Semenkova G.N., Kulahava T.A., Pinchuk S.V., Shadyro O.I. (2019). Cytotoxic and antiproliferative effects of thymoquinone on rat C6 glioma cells depend on oxidative stress. Mol. Cell. Biochem..

[124] Liou Y., Chen P., Chu S., Kao S., Chang Y., Hsieh Y., Chang H. (2019). Thymoquinone suppresses the proliferation of renal cell carcinoma cells via reactive oxygen species-induced apoptosis and reduces cell stemness. Environ. Toxicol..

[125] Butt A.S., Nisar N., Mughal T.A., Ghani N., Altaf I. (2019). Anti-oxidative and anti-proliferative activities of extracted phytochemical compound thymoquinone. J. Pak. Med. Assoc..

[126] Park J.E., Kim D.-H., Ha E., Choi S.M., Choi J.-S., Chun K.-S., Joo S.H. (2019). Thymoquinone induces apoptosis of human epidermoid carcinoma A431 cells through ROS-mediated suppression of STAT3. Chem. Interact..

[127] Hsu H.-H., Chen M.-C., Day C.H., Lin Y.-M., Li S.-Y., Tu C.-C., Padma V.V., Shih H.-N., Kuo W.-W., Huang C.-Y. (2017). Thymoquinone suppresses migration of LoVo human colon cancer cells by reducing prostaglandin E2 induced COX-2 activation. World J. Gastroenterol..

[128] Ulasli S.S., Celik S., Gunay E., Ozdemir M., Hazman O., Ozyurek A., Koyuncu T., Unlu M. (2013). Anticancer Effects of Thymoquinone, Caffeic Acid Phenethyl Ester and Resveratrol on A549 Non-small Cell Lung Cancer Cells Exposed to Benzo(a)pyrene. Asian Pac. J. Cancer Prev..

[129] Singh S.K., Apata T., Gordetsky J.B., Singh R. (2019). Docetaxel Combined with Thymoquinone Induces Apoptosis in Prostate Cancer Cells via Inhibition of the PI3K/AKT Signaling Pathway. Cancers.

[130] Fatfat M., Fakhoury I., Habli Z., Mismar R., Gali-Muhtasib H. (2019). Thymoquinone enhances the anticancer activity of doxorubicin against adult T-cell leukemia in vitro and in vivo through ROS-dependent mechanisms. Life Sci..

[131] Chen M.-C., Lee N.-H., Hsu H.-H., Ho T.-J., Tu C.-C., Hsieh D.J.-Y., Lin Y.-M., Chen L.-M., Kuo W.-W., Huang C.-Y. (2015). Thymoquinone Induces Caspase-Independent, Autophagic Cell Death in CPT-11-Resistant LoVo Colon Cancer via Mitochondrial Dysfunction and Activation of JNK and p38. J. Agric. Food Chem..

[132] Das S., Dey K.K., Dey G., Pal I., Majumder A., MaitiChoudhury S., Kundu S.C., Mandal M. (2012). Antineoplastic and Apoptotic Potential of Traditional Medicines Thymoquinone and Diosgenin in Squamous Cell Carcinoma. PLoS ONE.

[133] Hatiboglu M.A., Kocyigit A., Guler E.M., Akdur K., Khan I., Nalli A., Karatas E., Tuzgen S. (2019). Thymoquinone Enhances the Effect of Gamma Knife in B16-F10 Melanoma Through Inhibition of Phosphorylated STAT3. World Neurosurg..

[134] Samarghandian S., Azimi-Nezhad M., Farkhondeh T. (2019). Thymoquinone-induced antitumor and apoptosis in human lung adenocarcinoma cells. J. Cell. Physiol..

[135] Ndreshkjana B., Çapci A., Klein V., Chanvorachote P., Muenzner J., Huebner K., Steinmann S., Erlenbach-Wuensch K., Geppert C.I., Agaimy A. (2019). Combination of 5-fluorouracil and thymoquinone targets stem cell gene signature in colorectal cancer cells. Cell Death Dis..

[136] Lee S.-R., Mun J.-Y., Jeong M.-S., Lee H.-H., Roh Y.-G., Kim W.-T., Kim M.-H., Heo J., Choi Y.H., Kim S.J. (2019). Thymoquinone-Induced Tristetraprolin Inhibits Tumor Growth and Metastasis through Destabilization of MUC4 mRNA. Int. J. Mol. Sci..

[137] Santoso R.A., Lienaningrum A.S., Bangun E.D., Reformatika H.G., Susidarti R.A., Meiyanto E. Black cumin (*Nigella sativa* L.) and awar-awar (*Ficus septica burm*. F.) combination extract inhibits proliferation and modulates cell cycle on HeLa cell. Proceedings of the Fourth Huntsville Gamma-Ray Burst Symposium.

[138] Dera A., Rajagopalanz P. (2019). Thymoquinone attenuates phosphorylation of AKT to inhibit kidney cancer cell proliferation. J. Food Biochem..

[139] Lee Y.-M., Kim G.-H., Park E.-J., Oh T.-I., Lee S., Kan S.-Y., Kang H., Kim B.M., Kim J.H., Lim J.-H. (2019). Thymoquinone Selectively Kills Hypoxic Renal Cancer Cells by Suppressing HIF-1α-Mediated Glycolysis. Int. J. Mol. Sci..

[140] Butt A.S., Nisar N., Ghani N., Altaf I., Mughal T.A. (2019). Isolation of thymoquinone from Nigella sativa L. and Thymus vulgaris L., and its anti-proliferative effect on HeLa cancer cell lines. Trop. J. Pharm. Res..

[141] Khan A., Aldebasy Y.H., Alsuhaibani S.A., Khan M.A. (2019). Thymoquinone Augments Cyclophosphamide-Mediated Inhibition of Cell Proliferation in Breast Cancer Cells. Asian Pac. J. Cancer Prev..

[142] Suriyah W.H., Kasmuri A.R., Ni Foong F.H., Afriza D., Ichwan S.J.A. (2019). Comparison of the in vitro and in vivo toxic effects of thymoquinone using oral cancer HSC-3 and HSC-4 cell lines, oral fibroblasts, HACAT cell line, and Zebrafish embryos. Mater. Today Proc..

[143] Chaleshtori J.S., Heidari-Sureshjani E., Moradi F., Heidarian E. (2019). The Effects of Thymoquinone on Viability, and Anti-apoptotic Factors (BCL-XL, BCL-2, MCL-1) in Prostate Cancer (PC3) Cells: An In Vitro and Computer-Simulated Environment Study. Adv. Pharm. Bull..

[144] Sanjarin F., Sabouni F., Rashid M. (2019). Thymoquinone Effects on Cell Viability, Apoptosis and VEGF-A Gene Expression Level in AGS(CRL-1739) Cell Line. Anti-Cancer Agents Med. Chem..

[145] Ren X., Luo W. (2019). Exploration of pro-apoptotic effect of Thymoquinone on oral squamous cell carcinoma cells through PI3K/Akt signaling pathway. Cell. Mol. Biol..

[146] Zhang Y., Fan Y., Huang S., Wang G., Han R., Lei F., Luo A., Jing X., Zhao L., Gu S. (2018). Thymoquinone inhibits the metastasis of renal cell cancer cells by inducing autophagy via AMPK/mTOR signaling pathway. Cancer Sci..

[147] Bashmail H.A., AlAmoudi A.A., Noorwali A., Hegazy G.A., Ajabnoor G., Choudhry H., Al-Abd A.M. (2018). Thymoquinone synergizes gemcitabine anti-breast cancer activity via modulating its apoptotic and autophagic activities. Sci. Rep..

[148] Kou B., Kou Q., Ma B., Zhang J., Sun B., Yang Y., Li J., Zhou J., Liu W. (2018). Thymoquinone inhibits metastatic phenotype and epithelial-mesenchymal transition in renal cell carcinoma by regulating the LKB1/AMPK signaling pathway. Oncol. Rep..

[149] Zhang M., Du H., Huang Z., Zhang P., Yue Y., Wang W., Liu W., Zeng J., Ma J., Chen G. (2018). Thymoquinone induces apoptosis in bladder cancer cell via endoplasmic reticulum stress-dependent mitochondrial pathway. Chem. Interact..

[150] Subburayan K., Thayyullathil F., Pallichankandy S., Rahman A., Galadari S. (2018). Par-4-dependent p53 up-regulation plays a critical role in thymoquinone-induced cellular senescence in human malignant glioma cells. Cancer Lett..

[151] Khazaei M., Pazhouhi M., Sariri R., Khazaei M.R., Moradi M.T. (2018). Synergistic effect of temozolomide and thymoquinone on human glioblastoma multiforme cell line (U87MG). J. Cancer Res. Ther..

[152] Ibrahim A., Alhosin M., Papin C., Ouararhni K., Omran Z., Zamzami M.A., Al-Malki A.L., Choudhry H., Mély Y., Hamiche A. (2018). Thymoquinone challenges UHRF1 to commit auto-ubiquitination: A key event for apoptosis induction in cancer cells. Oncotarget.

[153] Hatiboglu M.A., Kocyigit A., Guler E.M., Akdur K., Nalli A., Karatas E., Tuzgen S. (2018). Thymoquinone Induces Apoptosis in B16-F10 Melanoma Cell Through Inhibition of p-STAT3 and Inhibits Tumor Growth in a Murine Intracerebral Melanoma Model. World Neurosurg..

[154] Öztürk E., Kaymak E., Akin A.T., Karabulut D., Ünsal H.M., Yakan B. (2020). Thymoquinone is a protective agent that reduces the negative effects of doxorubicin in rat testis. Hum. Exp. Toxicol..

[155] Alghamdi F., Al-Seeni M.N., Ghoneim M.A. (2020). Potential synergistic antioxidant effect of thymoquinone and vitamin E on cisplatin-induced acute nephropathy in rats. Clin. Nutr. Exp..

[156] Hossen M.J., Yang W.S., Kim D., Aravinthan A., Kim J.-H., Cho J.Y. (2017). Thymoquinone: An IRAK1 inhibitor with in vivo and in vitro anti-inflammatory activities. Sci. Rep..

[157] Özenver N., Efferth T. (2020). Small molecule inhibitors and stimulators of inducible nitric oxide synthase in cancer cells from natural origin (phytochemicals, marine compounds, antibiotics). Biochem. Pharmacol..

[158] Mosalam E.M., Zidan A.-A.A., Mehanna E.T., Mesbah N.M., Abo-Elmatty D.M. (2020). Thymoquinone and pentoxifylline enhance the chemotherapeutic effect of cisplatin by targeting Notch signaling pathway in mice. Life Sci..

[159] Alabdullah S.W., Alsamir S.A., AlRufaei I.A. (2020). Effect of Thymoquinone on some biochemical and hormonal indices and their protective effect on the genital organs of rats after cancer induction in Laboratory. EurAsian J. BioSci..

[160] Helmy S.A., El-Mesery M., El-Karef A., Eissa L.A., El Gayar A.M. (2019). Thymoquinone upregulates TRAIL/TRAILR2 expression and attenuates hepatocellular carcinoma in vivo model. Life Sci..

[161] Shahin Y., Elguindy N., Abdel Bary A., Balbaa M. (2018). The protective mechanism of Nigella sativa against diethylnitrosamine-induced hepatocellular carcinoma through its antioxidant effect and EGFR/ERK1/2 signaling. Environ. Toxicol..

[162] Pu Y., Hu S., Chen Y., Zhang Q., Xia C., Deng H., Wang Y., Hu Q. (2021). Thymoquinone loaded calcium alginate and polyvinyl alcohol carrier inhibits the 7,12-dimethylbenz[a]anthracene-induced hamster oral cancer via the down-regulation of PI3K/AKT/mTOR signaling pathways. Environ. Toxicol..

[163] Abdelbaky N.W., Abdelazem A.Z., Hashem K.S. (2020). Thymoquinone Attenuates 6-Mercaptopurine Induced Testicular Toxicity in Albino Rats: Possible Mechanisms are Involved. Adv. Anim. Vet. Sci..

[164] Pal R.R., Parashar P., Singh I., Saraf S.A. (2019). Tamanu oil potentiated novel sericin emulgel of levocetirizine: Repurposing for topical delivery against DNCB-induced atopic dermatitis, QbD based development and in vivo evaluation. J. Microencapsul..

[165] Pal R.R., Maurya A.K., Parashar P., Saraf S.A. (2020). A Comparative Study of Levocetirizine Loaded Vesicular and Matrix Type System for Topical Application: Appraisal of Therapeutic Potential against Atopic Dermatitis. J. Pharm. Innov..

[166] Kalyane D., Raval N., Maheshwari R., Tambe V., Kalia K., Tekade R.K. (2019). Employment of enhanced permeability and retention effect (EPR): Nanoparticle-based precision tools for targeting of therapeutic and diagnostic agent in cancer. Mater. Sci. Eng. C Mater. Biol. Appl..

[167] Zhao J., Chen X., Ho K.-H., Cai C., Li C.-W., Yang M., Yi C. (2021). Nanotechnology for diagnosis and therapy of rheumatoid arthritis: Evolution towards theranostic approaches. Chin. Chem. Lett..

[168] Maurya P., Singh S., Mishra N., Pal R., Singh N., Parashar P., Saraf S.A., Bera H., Hossain C.M., Saha S. (2021). Chapter 20—Albumin-based nanomaterials in drug delivery and biomedical applications. Biopolymer-Based Nanomaterials in Drug Delivery and Biomedical Applications.

[169] Golombek S.K., May J.-N., Theek B., Appold L., Drude N., Kiessling F., Lammers T. (2018). Tumor targeting via EPR: Strategies to enhance patient responses. Adv. Drug Deliv. Rev..

[170] Zafar S., Akhter S., Ahmad I., Hafeez Z., Alam Rizvi M.M., Jain G.K., Ahmad F.J. (2020). Improved chemotherapeutic efficacy against resistant human breast cancer cells with co-delivery of Docetaxel and Thymoquinone by Chitosan grafted lipid nanocapsules: Formulation optimization, in vitro and in vivo studies. Colloids Surf. B Biointerfaces.

[171] Odeh F., Naffa R., Azzam H., Mahmoud I.S., Alshaer W., Al Bawab A., Ismail S. (2019). Co-encapsulation of thymoquinone with docetaxel enhances the encapsulation efficiency into PEGylated liposomes and the chemosensitivity of MCF7 breast cancer cells to docetaxel. Heliyon.

[172] Bhatt P., Lalani R., Vhora I., Patil S., Amrutiya J., Misra A., Mashru R. (2018). Liposomes encapsulating native and cyclodextrin enclosed paclitaxel: Enhanced loading efficiency and its pharmacokinetic evaluation. Int. J. Pharm..

[173] Zafar S., Akhter S., Garg N., Selvapandiyan A., Jain G.K., Ahmad F.J. (2020). Co-encapsulation of docetaxel and thymoquinone in mPEG-DSPE-vitamin E TPGS-lipid nanocapsules for breast cancer therapy: Formulation optimization and implications on cellular and in vivo toxicity. Eur. J. Pharm. Biopharm..

[174] Ahmad R., Kaus N.H.M., Hamid S. (2020). Synthesis and characterization of PLGA-PEG thymoquinone nanoparticles and its cytotoxicity effects in tamoxifen-resistant breast cancer cells. Cancer Biology and Advances in Treatment.

[175] Kumar V.S., Devi R.P., Hemananthan E. (2019). In vitro studies to analyze the stability and bioavailability of thymoquinone encapsulated in the developed nanocarrier. J. Dispers. Sci. Technol..

[176] Kommineni N., Saka R., Bulbake U., Khan W. (2019). Cabazitaxel and thymoquinone co-loaded lipospheres as a synergistic combination for breast cancer. Chem. Phys. Lipids.

[177] Borodina T., Gileva A., Akasov R., Trushina D., Burov S., Klyachko N., González-Alfaro Y., Bukreeva T., Markvicheva E. (2021). Fabrication and evaluation of nanocontainers for lipophilic anticancer drug delivery in 3D in vitro model. J. Biomed. Mater. Res. Part B Appl. Biomater..

[178] Goel S., Mishra P. (2019). Thymoquinone loaded mesoporous silica nanoparticles retard cell invasion and enhance in vitro cytotoxicity due to ROS mediated apoptosis in HeLa and MCF-7 cell lines. Mater. Sci. Eng. C.

[179] Shahein S.A., Aboul-Enein A.M., Higazy I.M., Abou-Elella F., Lojkowski W., Ahmed E.R., Mousa S.A., AbouAitah K. (2019). Targeted anticancer potential against glioma cells of thymoquinone delivered by mesoporous silica core-shell nanoformulations with pH-dependent release. Int. J. Nanomed..

[180] AlShehri S., Imam S.S., Rizwanullah M., Fakhri K.U., Alam Rizvi M.M., Mahdi W., Kazi M. (2020). Effect of Chitosan Coating on PLGA Nanoparticles for Oral Delivery of Thymoquinone: In Vitro, Ex Vivo, and Cancer Cell Line Assessments. Coatings.

[181] Sharifi F., Yesil-Celiktas O., Kazan A., Maharjan S., Saghazadeh S., Firoozbakhsh K., Firoozabadi B., Zhang Y.S. (2020). A hepatocellular carcinoma–bone metastasis-on-a-chip model for studying thymoquinone-loaded anticancer nanoparticles. Bio-Des. Manuf..

[182] Azmy N., Haron A.S., Alwi S.S.S. (2019). Thymoquinone-loaded nanostructured lipid carrier reduces proliferation of human liver cancer cells, HepG2. Malays. J. Med. Health Sci..

[183] Sunoqrot S., Alfaraj M., Hammad A., Kasabri V., Shalabi D., Deeb A., Ibrahim L.H., Shnewer K., Yousef I. (2020). Development of a Thymoquinone Polymeric Anticancer Nanomedicine through Optimization of Polymer Molecular Weight and Nanoparticle Architecture. Pharmaceutics.

[184] Alhakamy N.A., Badr-Eldin S.M., A Fahmy U., Alruwaili N.K., Awan Z.A., Caruso G., Alfaleh M.A., Alaofi A.L., Arif F.O., Ahmed O.A. (2020). Thymoquinone-Loaded soy-phospholipid-based phytosomes exhibit anticancer potential against human lung cancer cells. Pharmaceutics.

[185] Bergonzi M.C., Vasarri M., Marroncini G., Barletta E., Degl’Innocenti D. (2020). Thymoquinone-Loaded Soluplus^®^-Solutol^®^ HS15 Mixed Micelles: Preparation, In Vitro Characterization, and Effect on the SH-SY5Y Cell Migration. Molecules.

[186] Ibiyeye K.M., Zuki A.B.Z. (2020). Cockle Shell-Derived Aragonite CaCO3 Nanoparticles for Co-Delivery of Doxorubicin and Thymoquinone Eliminates Cancer Stem Cells. Int. J. Mol. Sci..

[187] Mehanna M.M., Sarieddine R., Alwattar J.K., Chouaib R., Gali-Muhtasib H. (2020). Anticancer Activity of Thymoquinone Cubic Phase Nanoparticles Against Human Breast Cancer: Formulation, Cytotoxicity and Subcellular Localization. Int. J. Nanomed..

[188] Ibrahim W.N., Rosli L.M.B.M., Doolaanea A.A. (2020). Formulation, Cellular Uptake and Cytotoxicity of Thymoquinone-Loaded PLGA Nanoparticles in Malignant Melanoma Cancer Cells. Int. J. Nanomed..

[189] Vhora I., Patil S., Bhatt P., Gandhi R., Baradia D., Misra A. (2014). Receptor-targeted drug delivery: Current perspective and challenges. Ther. Deliv..

[190] Caveliers V., Everaert H., John C.S., Lahoutte T., Bossuyt A. (2002). Sigma receptor scintigraphy with N-[2-(1′-piperidinyl)ethyl]-3-(123)I-iodo-4-methoxybenzamide of patients with suspected primary breast cancer: First clinical results. J. Nucl. Med..

[191] Banerjee R., Tyagi P., Li S., Huang L. (2004). Anisamide-targeted stealth liposomes: A potent carrier for targeting doxorubicin to human prostate cancer cells. Int. J. Cancer.

[192] Ramzy L., Metwally A.A., Nasr M., Awad G.A.S. (2020). Novel thymoquinone lipidic core nanocapsules with anisamide-polymethacrylate shell for colon cancer cells overexpressing sigma receptors. Sci. Rep..

[193] Singh S.K., Gordetsky J.B., Bae S., Acosta E.P., Lillard J.J.W., Singh R. (2020). Selective Targeting of the Hedgehog Signaling Pathway by PBM Nanoparticles in Docetaxel-Resistant Prostate Cancer. Cells.

[194] Bhattacharya S., Ghosh A., Maiti S., Ahir M., Debnath G.H., Gupta P., Bhattacharjee M., Ghosh S., Chattopadhyay S., Mukherjee P. (2020). Delivery of thymoquinone through hyaluronic acid-decorated mixed Pluronic^®^ nanoparticles to attenuate angiogenesis and metastasis of triple-negative breast cancer. J. Control. Release.

[195] Ince I., Yıldırım Y., Güler G., Medine E.I., Ballıca G., Kuşdemir B.C., Göker E. (2020). Synthesis and characterization of folic acid-chitosan nanoparticles loaded with thymoquinone to target ovarian cancer cells. J. Radioanal. Nucl. Chem..

[196] Upadhyay P., Sarker S., Ghosh A., Gupta P., Das S., Ahir M., Bhattacharya S., Chattopadhyay S., Ghosh S., Adhikary A. (2019). Transferrin-decorated thymoquinone-loaded PEG-PLGA nanoparticles exhibit anticarcinogenic effect in non-small cell lung carcinoma via the modulation of miR-34a and miR-16. Biomater. Sci..

[197] Murphy E.M., Centner C.S., Bates P.J., Malik M.T., Kopechek J.A. (2020). Delivery of thymoquinone to cancer cells with as1411-conjugated nanodroplets. PLoS ONE.

[198] Reyes-Reyes E.M., Teng Y., Bates P.J. (2010). A New Paradigm for Aptamer Therapeutic AS1411 Action: Uptake by Macropinocytosis and Its Stimulation by a Nucleolin-Dependent Mechanism. Cancer Res..

[199] Tokunaga E., Kimura Y., Mashino K., Oki E., Kataoka A., Ohno S., Morita M., Kakeji Y., Baba H., Maehara Y. (2006). Activation of PI3K/Akt signaling and hormone resistance in breast cancer. Breast Cancer.

[200] Rajput S., Puvvada N., Kumar B.N.P., Sarkar S., Konar S., Bharti R., Dey G., Mazumdar A., Pathak A., Fisher P.B. (2015). Overcoming Akt Induced Therapeutic Resistance in Breast Cancer through siRNA and Thymoquinone Encapsulated Multilamellar Gold Niosomes. Mol. Pharm..

[201] Kumar S.R., Thangam R., Vivek R., Srinivasan S., Ponpandian N. (2020). Synergetic effects of thymoquinone-loaded porous PVPylated Fe_3_O_4_ nanostructures for efficient pH-dependent drug release and anticancer potential against triple-negative cancer cells. Nanoscale Adv..

[202] Sweety J.P., Sowparani S., Mahalakshmi P., Selvasudha N., Yamini D., Geetha K., Ruckmani K. (2020). Fabrication of stimuli gated nanoformulation for site-specific delivery of thymoquinone for colon cancer treatment—Insight into thymoquinone’s improved physicochemical properties. J. Drug Deliv. Sci. Technol..

[203] Tariq S., Naqvi S.A.R., Naz S., Mubarik M.S., Yaseen M., Riaz M., Shah S.M.A., Rafi M., Roohi S. (2020). Dose-Dependent Internalization and Externalization Integrity Study of Newly Synthesized 99mTc-Thymoquinone Radiopharmaceutical as Cancer Theranostic Agent. Dose-Response.

[204] Fahmy H.M. (2019). In vitro study of the cytotoxicity of thymoquinone/curcumin fluorescent liposomes. Naunyn-Schmiedeberg’s Arch. Pharmacol..

[205] Fathy M.M. (2020). Multifunctional Thymoquinone-Capped Iron Oxide Nanoparticles for Combined Chemo-Photothermal Therapy of Cancer. J. Supercond. Nov. Magn..

[206] Das S., Bera D., Pal K., Mondal D., Karmakar P., Das S., Dey A. (2020). Guar gum micro-vehicle mediated delivery strategy and synergistic activity of thymoquinone and piperine: An in vitro study on bacterial and hepatocellular carcinoma cells. J. Drug Deliv. Sci. Technol..

[207] Alkhatib M.H., Bawadud R.S., Gashlan H.M. (2020). Incorporation of docetaxel and thymoquinone in borage nanoemulsion potentiates their antineoplastic activity in breast cancer cells. Sci. Rep..

[208] Altamimi M.A., Kazi M., Albgomi M.H., Ahad A., Raish M. (2019). Development and optimization of self-nanoemulsifying drug delivery systems (SNEDDS) for curcumin transdermal delivery: An anti-inflammatory exposure. Drug Dev. Ind. Pharm..

[209] Rushmi Z.T., Akter N., Mow R.J., Afroz M., Kazi M., de Matas M., Rahman M., Shariare M.H. (2017). The impact of formulation attributes and process parameters on black seed oil loaded liposomes and their performance in animal models of analgesia. Saudi Pharm. J..

[210] Kazi M., A Nasr F., Noman O., Alharbi A., Alqahtani M.S., Alanazi F.K. (2020). Development, Characterization Optimization, and Assessment of Curcumin-Loaded Bioactive Self-Nanoemulsifying Formulations and Their Inhibitory Effects on Human Breast Cancer MCF-7 Cells. Pharmaceutics.

[211] Guria S., Ghosh A., Upadhyay P., Das M.K., Mishra T., Adhikary A., Adhikari S. (2020). Small-Molecule Probe for Sensing Serum Albumin with Consequential Self-Assembly as a Fluorescent Organic Nanoparticle for Bioimaging and Drug-Delivery Applications. ACS Appl. Bio Mater..

[212] Khattabi A.M., Alqdeimat D.A., Sabbar E., Talib W.H. (2020). In Vitro Characteristics of a Combination of Thymoquinone-Resveratrol Loaded and Targeted Nanodrug Delivery System. Jordan J. Pharm. Sci..

[213] Dawaba A.M., Dawaba H.M. (2020). Application of Optimization Technique to Develop Nano-Based Carrier of Nigella Sativa Essential Oil: Characterization and Assessment. Recent Pat. Drug Deliv. Formul..

[214] Kaus N.H.M., Shaarani S., Hamid S.S. (2017). The Influence of pluronic F68 and F127 nanocarrier on physicochemical properties, in vitro release, and antiproliferative activity of thymoquinone drug. Pharmacogn. Res..

[215] Fakhoury I., Saad W., Bouhadir K., Nygren P., Schneider-Stock R., Gali-Muhtasib H. (2016). Uptake, delivery, and anticancer activity of thymoquinone nanoparticles in breast cancer cells. J. Nanopart. Res..

[216] Akasov R., Borodina T., Zaytseva E., Sumina A., Bukreeva T., Burov S., Markvicheva E. (2015). Ultrasonically Assisted Polysaccharide Microcontainers for Delivery of Lipophilic Antitumor Drugs: Preparation and in Vitro Evaluation. ACS Appl. Mater. Interfaces.

[217] Dehghani H., Hashemi M., Entezari M., Mohsenifar A. (2015). The Comparison of Anticancer Activity of Thymoquinone and Nanothymoquinone on Human Breast Adenocarcinoma. Iran. J. Pharm. Res. IJPR.

[218] El-Toni A.M., Khan A., Ibrahim M.A., Labis J.P., Badr G., Al-Hoshan M., Yin S., Sato T. (2012). Synthesis of double mesoporous core–shell silica spheres with tunable core porosity and their drug release and cancer cell apoptosis properties. J. Colloid Interface Sci..

[219] Ganea G.M., Fakayode S.O., Losso J.N., Van Nostrum C.F., Sabliov C.M., Warner I.M. (2010). Delivery of phytochemical thymoquinone using molecular micelle modified poly(D, L lactide-co-glycolide) (PLGA) nanoparticles. Nanotechnology.

[220] Fathy M.M. (2019). Biosynthesis of Silver Nanoparticles Using Thymoquinone and Evaluation of Their Radio-Sensitizing Activity. BioNanoSci..

[221] Yazan L.S., Mohd Azlan S., Zakarial Ansar F., Gopalsamy B. (2019). Acute toxicity study of intravenous administration of thymoquinone-loaded nanostructured lipid carrier (TQ-NLC) in Sprague Dawley rats. Malays. J. Med. Health Sci..

[222] Khader M., Bresgen N., Eckl P. (2009). In vitro toxicological properties of thymoquinone. Food Chem. Toxicol..

[223] Kassab R.B., El-Hennamy R.E. (2017). The role of thymoquinone as a potent antioxidant in ameliorating the neurotoxic effect of sodium arsenate in female rat. Egypt. J. Basic Appl. Sci..

[224] Yahyazadeh A., Altunkaynak B.Z. (2019). Investigation of the neuroprotective effects of thymoquinone on rat spinal cord exposed to 900 MHz electromagnetic field. J. Chem. Neuroanat..

[225] Abuzinadah M.F., Ahmad A. (2019). Pharmacological studies on the efficacy of a thymoquinone-containing novel polyherbal formulation against cisplatin-induced hepatorenal toxicity in rats. J. Food Biochem..

[226] Crede P. (2014). Treatment of Inflammatory Disease or Disorder and Compositions Therefor. U.S. Patent.

[227] Özen O.A., Özgül M., Aydin M. (2016). Nanomicelles for the Treatment of Cancer.

[228] Malik M.T., Kopechek J.A., Bates P.J. (2019). Targeted Nanodroplet Emulsions for Treating Cancer. U.S. Patent.

[229] Halwani M.A., Balkhy H.H. (2016). Nano-Liposomal Aminoglycoside-Thymoquinone Formulations.

[230] Salem A.E., El Haty I., Abdou I., Adem A., Attoub S. (2019). Thymoquinone Derivatives for Treatment of Cancer. U.S. Patent.

[231] Poonam N., Charul R., Sharma I.S. (2018). Improved Vesicular Formulation of Thymoquinone for the Treatment of Dermal Inflammatory Disorders and Method Thereof.

[232] Crede P. (2013). Compositions Comprising Thymoquinone for the Treatment of Inflammatory Diseases.

[233] Odeh F., Ismail S. (2016). Liposomal Formulations Comprising Thymoquinone and Taxane, and Method of Treating Cancer Using Same.

[234] Zhu N., Xiang Y., Zhao X., Cai C., Chen H., Wenbing J., Wang Y., Zeng C. (2019). Application of the Thymoquinone in Preparation Prevention Intravascular Stent Restenosis Medicaments.

[235] Madhavamenon K.I., Maliakel B.P., Ittiyavirah S.P., Ramalingam K. (2019). Composition of Nigella Sativaseeds to Treat Anxiety, Stress and Sleep Disorders with Significant Memory Enhancement Properties and a Process for Producing the Same. U.S. Patent.

[236] Fazlul H.S., Ramzi M.M. (2011). Thymoquinone Analogs for the Treatment of Pancreatic Cancer.

[237] Crooks P.A., Worthen D.R., Ghosheh O.A. (2001). Use of the Naturally-Occurring Quinones Thymoquinone and Dithymoquinone as Antineoplastic and Cytotoxic Agents. U.S. Patent.

[238] Wan X., Wu H. (2013). Synthesis Method of Thymoquinone Serving as Blood Vessel Inhibition Medicament.

[239] Jianping Z., Ma Z., Lifa Z. (2014). Hyaluronic Acid-Adipodihydrazide-Thymoquinone Grafted Polymer as Well as Synthesis Method and Application of Hyaluronic Acid-Adipodihydrazide-Thymoquinone Grafted Polymer.

[240] Pacioretty L., Babish J. (2011). Formulations Containing Thymoquinone For Urinary Health. U.S. Patent.

[241] Mekkawi S.S.R. (1998). Use of Iron-Binding Glycoproteins and/or 10-Hydroxy-2-Decenoic Acid in Combination with Thymoquinone for Treating Immunodeficiency Diseases.

